# Synthesis and antibacterial activities of enamine derivatives of dehydroacetic acid

**DOI:** 10.1007/s00044-017-2110-8

**Published:** 2017-11-13

**Authors:** Alex G. Baldwin, Jonathan Bevan, David Brough, Ruth Ledder, Sally Freeman

**Affiliations:** 1 Division of Pharmacy and Optometry, School of Health Sciences, Faculty of Biology, Medicine and Health, Manchester Academic Health Science Centre, The University of Manchester, Stopford Building, Oxford Road, Manchester, M13 9PT UK; 2 Division of Neuroscience and Experimental Psychology, School of Biological Sciences, Faculty of Biology, Medicine and Health, Manchester Academic Health Science Centre, The University of Manchester, AV Hill Building, Oxford Road, Manchester, M13 9PT UK

**Keywords:** Dehydroacetic acid, Pyran-2, 4-diones, *Staphylococcus aureus*, SAR, Antibacterial activity

## Abstract

Dehydroacetic acid is a common pyrone derivative used commercially as an antibacterial and antifungal agent. Based on the synthesis of dehydroacetic acid (**1**) from *N*-hydroxysuccinimdyl acetoacetate, a novel series of enamine-based derivatives were synthesised in order to improve the antibacterial activity of dehydroacetic acid. The antibacterial activities of the synthesised analogues were evaluated against *Escherichia coli* and *Staphylococcus aureus*. Derivative **4d** (N-Ph) was identified as the most potent inhibitor of *S. aureus* growth. Overall, derivative **4b** (N-Me) showed the best broad-spectrum activity with five-fold greater minimum inhibitory concentration and 11-fold greater minimum biocidal concentration against *E. coli* when compared to dehydroacetic acid, in addition to improved antibacterial activity against *S. aureus*.

## Introduction

There is an urgent need for the development of new antibacterial agents in order to overcome bacterial resistance. A number of clinically important bacterial pathogens including *Staphylococcus aureus* that were once susceptible to antibiotic therapy are rapidly becoming multidrug-resistant due to misuse of antibacterial agents within the medical and agricultural field (Davies and Davies 2010; Blair et al. 2015). Recently, resistance to the last-resort antibiotic colistin has emerged in animals and humans that have spread globally from China, resulting in the potential for pan-resistance among pathogenic bacteria (Liu et al. 2016).

3-Acetyl-4-hydroxy-6-methyl-2*H*-pyran-2-one (dehydroacetic acid) (**1**), and its derivatives are known to possess antibacterial, antifungal and phytotoxic activity (Nagawade et al. 2005; Dias et al. 2009; Pulate et al. 2011; Nechak et al. 2015). **1** or its salt sodium dehydroacetate, are safe and are widely found in cosmetics and personal care products as well as additives in food and drink. **1** is also a versatile starting material for the preparation of a variety of heterocycles and cycloaddition products (Goel and Ram 2009; Fadda and Elattar 2016).

Our intention was to synthesise acetoacetamide derivatives (**3**) by reacting commercially available *N*-hydroxysuccinimidyl acetoacetate (**2**) with a range of amines (R = H, Me, Et, Ph and substituted anilines, CH_2_Ph, CH_2_CH_2_Ph, cyclohexyl and CH_2_C≡CH). These were required as intermediates for a novel class of anti-inflammatory agents that target the NLRP3 inflammasome (Baldwin et al. 2017). However, surprisingly it soon became apparent following characterisation of the products that the desired compounds (**3**) were not obtained and that the enamine analogues (**4**) were synthesised, arising from **1**. Herein, we discuss the novel synthesis of **1** from **2** and given its known antibacterial activity, a number of enamine analogues (**4**) were synthesised in order to improve its antibacterial properties.

## Materials and methods

### Experimental

A batch of *N*-hydroxysuccinimidyl acetoacetate (purchased from Acros Organics) was characterised as dehydroacetic acid. Solvents were purchased from Fisher Scientific. All other chemicals were purchased from Sigma-Aldrich. A Bruker Avance 300/400 spectrometer was used to record ^1^H and ^13^C NMR spectra at ambient temperature. Chemical shifts are defined in ppm (*δ*) relative to CHCl_3_ (*δ* = 7.26 ppm). Electrospray ionisation mass spectrometry (ESI-MS) and high resolution-mass spectrometry (HRMS) were carried out on Waters SQD2 or Waters QTOF instruments with lock spray. Infrared spectroscopy was conducted on a JASCO FT/IR-4100 spectrophotometer using the Spectra Manager II (JASCO) software package. Melting points were taken using a Stuart SMP10 digital melting point apparatus. Evaporation of solvents was conducted on a Buchi Rotavapor R-200 equipped with Buchi heating bath B-490. TLC was performed using silica gel 60 on aluminium sheets with F_254_. All spots were visualised using a MV Mineralight lamp (254/365) UVGL-58. Silica gel with particle size 40–63 microns was used for column chromatography. All purified products using the column chromatography method were evaporated in vacuo to completeness. Full characterisation data for the novel compounds (**4f–i** and **4k–l**) is given below and the ^1^H/^13^C NMR data are shown in Table 1. Full characterisation data for compounds that have been reported before (**4a–e** and **4j**) are given in the Supplementary Information (SI). All spectra for compounds **4a**–**4l** are included in the Supplementary Information.

**Table 1:** ^1^H and ^13^C NMR data for dehydroacetic acid analogues **4f**–**4i** and **4k**–**4l** in CDCl_3_

Compound	^1^H NMR (*δ*, ppm)	^13^C NMR (*δ*, ppm)
**4f** ^**a**^	14.27 (1H, br s, NH), 7.18–7.38 (5H, m, Ph), 5.68 (1H, s, H5), 3.72 (2H, dt ~ q, *J* = 6.5 Hz, H1’), 3.00 (2H, t, *J* = 7.2 Hz, H2’), 2.51 (3H, s, CH_3_), 2.11 (3H, s, CH_3_)	184.6 (C8), 176.0 (C4), 163.8 (C2), 162.7 (C6), 137.1 (C3’), 128.9 (C5’ and C7’), 128.7 (C4’ and C8’), 127.2 (C6’), 107.4 (C5), 96.5 (C3), 45.7 (C1’), 35.6 (C2’), 19.8 (C9), 18.0 (C7)
**4g** ^**b**^	14.25 (1H, br s, NH), 5.67 (1H, s, H5), 3.66–3.78 (1H, m, H1’), 2.64 (3H, s, CH_3_), 2.12 (3H, s, CH_3_), 1.88–1.97 (2H, m, H2’_eq_ and H6’_eq_), 1.76–1.85 (2H, m, H3’_eq_ and H5’_eq_), 1.24–1.57 (6H, m, H2’_ax_, H3’_ax_, H4’, H5’_ax_ and H6’_ax_)	184.8 (C8), 174.3 (C4), 162.7 (C6), 107.6 (C5), 96.4 (C3), 52.9 (C1’), 32.8 (C2’ and C6’), 25.2 (C3’ and C5’), 24.1 (C4’), 19.9 (C9), 18.3 (C7), C2 signal not observed.
**4h** ^**a**^	14.40 (1H, br s, NH), 5.70 (1H, s, H5), 4.23 (2H, dd, *J* = 2.4 and 5.4 Hz, H1’), 2.71 (3H, s, CH_3_), 2.40 (1H, t, *J* = 2.4 Hz, H3’), 2.12 (3H, s, CH_3_)	184.8 (C8), 177.1 (C4), 163.5 (C2), 163.1 (C6), 107.3 (C5), 97.2 (C3), 76.4 (C2’), 74.0 (C3’), 33.4 (C1’), 19.9 (C9), 18.3 (C7)
**4i** ^**a**^	7.32–7.45 (2H, m, H4’ and H5’), 7.20 (1H, s, H2’), 7.08 (1H, d, *J* = 7.2 Hz, H6’), 5.76 (1H, s, H5’), 2.59 (3H, s, CH_3_), 2.17 (3H, s, CH_3_)	185.1 (C8), 175.7 (C4), 163.9 (C2), 163.4 (C6), 137.7 (C1’), 135.4 (C3’), 130.7 (C5’), 128.4 (C2’), 126.0 (C6’), 124.1 (C4’), 107.2 (C5), 97.7 (C3), 20.5 (C9), 20.1 (C7)
**4k** ^**a**^	7.56–7.70 (2H, m, H4’ and H5’), 7.46 (1H, s, H2’), 7.38 (1H, d, *J* = 7.2 Hz, H6’), 5.78 (1H, s, H5), 2.61 (3H, s, CH_3_), 2.18 (3H, s, CH_3_)	185.2 (C8), 175.7 (C4), 164.1 (C2), 163.3 (C6), 137.3 (C1’), 132.5 (q, *J*_CF_ = 33.0 Hz, C3’), 130.5 (C5’), 129.2 (C6’), 125.0 (q, *J*_CF_ = 3.7 Hz, C2’), 123.4 (*J*_CF_ = 270.9 Hz, CF_3_), 122.8 (*J*_CF_ = 3.75 Hz, C4’), 107.1 (C5), 97.9 (C3), 20.4 (C9), 20.1 (C7)
**4l** ^**a**^	7.46 (2H, d, *J* = 8.4 Hz, H3’ and H5’), 7.10 (2H, d, *J* = 8.7 Hz, H2’ and H6’), 5.76 (1H, s, H5), 2.60 (3H, s, CH_3_), 2.17 (3H, s, CH_3_), 1.34 (9H, s, C(CH_3_)_3_)	185.1 (C8), 175.5 (C4), 163.7 (C2), 163.4 (C6), 151.5 (C4’), 133.8 (C1’), 126.7 (C3’ and C5’), 125.2 (C2’ and C6’), 107.4 (C5), 97.5 (C3), 34.9 (C7’), 31.4 (C8’), 20.5 (C9), 20.1 (C7)

^a^ 300/75 MHz spectrometer

^b^ 400/100 MHz spectrometer

### General procedure for synthesis of pyran-2,4(3*H*)-diones

Unless otherwise stated, dehydroacetic acid (**1**) (0.20 g, 1.19 mmol) in anhydrous dichloromethane (DCM) (5 mL) was added dropwise to a solution containing the primary amine (1.19 mmol) and Et_3_N (0.33 mL, 2.38 mmol) in anhydrous DCM (5 mL) under N_2_. The reaction was stirred at room temperature and monitored by TLC until completion. Upon reaction completion, DCM (10 mL) was added to the reaction mixture and washed with 5% HCl (2 × 10 mL). The extracted organic layer was dried over MgSO_4_, filtered, and the reaction mixture was purified by flash column chromatography (EtOAc:*n*-hexane) to obtain the pure pyran-2,4-dione derivative. Ratio of solvents used for flash column chromatography is stated for each compound.

#### (*E*)-6-Methyl-3-(1-(phenethylamino)ethylidene)-2*H*-pyran-2,4(3 *H*)-dione (**4f**)

Cream solid. EtOAc:*n*-hexane (1:3). Yield: 93%; mp 89–90 °C; IR (neat): *ν*_max_ 1698, 1654, 1571, 1458, 1360, 1331, 997 cm^−1^; MS (ESI^+^) (*m*/*z*): 272.1 [M+H, 100]^+^, 294.1 [M+Na, 10]^+^; HRMS (APCI^+^ TOF-MS) (*m*/*z*): [M+H]^+^ calcd. for C_16_H_18_NO_3_, 272.1287, found: 272.1277, error: 3.7 ppm.

#### (*E*)-3-(1-(Cyclohexylamino)ethylidene)-6-methyl-2*H*-pyran-2,4(3*H*)-dione (**4g**)

Cream solid. EtOAc:*n*-hexane (1:3). Yield: 56%; mp 109–110 °C; IR (neat): *ν*_max_ 2933, 2857, 1696, 1668, 1559, 1473, 1353, 1001 cm^−1^; MS (ESI^+^) (*m*/*z*): 250.2 [M+H, 100]^+^, 272.2 [M+Na, 38]^+^; HRMS (APCI^+^ TOF-MS) (*m*/*z*): [M+H]^+^ calcd. for C_14_H_20_NO_3_, 250.1443, found: 250.1434, error: 3.6 ppm.

#### (*E*)-6-Methyl-3-(1-(prop-2-yn-1-ylamino)ethylidene)-2*H*-pyran-2,4(3*H*)-dione (**4h**)

Cream solid. EtOAc:*n*-hexane (2:3). Yield: 50%; mp 114–115 °C; IR (neat): *ν*_max_ 3241, 1694, 1661, 1581, 1466, 1357, 1321, 1000 cm^−1^; MS (ESI^+^) (*m*/*z*): 206.0 [M+H, 100]^+^; HRMS(APCI^+^ TOF-MS) (*m*/*z*): [M+H]^+^ calcd. for C_11_H_12_NO_3_, 206.0817, found: 206.0824, error: 3.4 ppm.

#### (*E*)-3-(1-(3-Chlorophenylamino)ethylidene)-6-methyl-2*H*-pyran-2,4(3*H*)-dione (**4i**)

The reaction was refluxed in 1,2-dichloroethane (DCE) instead of DCM at room temperature. Orange solid. EtOAc:*n*-hexane (1:4). Yield: 21%. mp 133–134 °C; MS (ESI^+^) (*m*/*z*): 278.1 [M+H, ^35^Cl, 100]^+^, 280.1 [M+H, ^37^Cl, 33]^+^, 300.1 [M+Na, ^35^Cl, 12]^+^, 302.0 [M+Na, ^37^Cl, 5]^+^; HRMS (ESI^+^) (*m*/*z*): [M+H]^+^ calcd. for C_14_H_13_^35^ClNO_3_, 278.0578, found: 278.0578, error: 0.0 ppm.

#### (*E*)-6-Methyl-3-(1-((3-(trifluoromethyl)phenyl)amino)ethylidene)-2*H*-pyran-2,4(3*H*)-dione (**4k**)

The reaction was refluxed in DCE instead of DCM at room temperature. Cream solid. EtOAc:*n*-hexane (1:4). Yield: 35%. mp 145–146 °C; MS (ESI^+^) (*m*/*z*): 312.2 [M+H, 82]^+^, 334.1 [M+Na, 12]^+^; HRMS (ESI^+^) (*m*/*z*): [M+H]^+^ calcd. for C_15_H_13_F_3_NO_3_, 312.0842, found: 312.0839, error: 1.0 ppm.

#### (*E*)-3-(1-((4-(*tert*-Butyl)phenyl)amino)ethylidene)-6-methyl-2*H*-pyran-2,4(3*H*)-dione (**4l**)

The reaction was refluxed in DCE instead of DCM at room temperature. Cream solid. EtOAc:*n*-hexane (1:4). Yield: 34%. mp 163–164 °C; MS (ESI^+^) (*m*/*z*): 300.2 [M+H, 100]^+^, 322.2 [M+Na, 5]^+^; HRMS (ESI^+^) (*m*/*z*): [M+H]^+^ calcd. for C_18_H_22_NO_3_, 300.1594, found: 300.1602, error: 2.7 ppm.

### Assay for antibacterial activity

The stock solutions of all test compounds (10.2–22.6 mg) were prepared by dissolving in absolute ethanol (1 mL). Following this they were filtered sterilised (0.2 micron pore size) to remove potential contaminants. Minimum inhibitory concentrations (MICs) and minimum biocidal concentrations (MBCs) were carried out against *Escherichia coli* ATCC 25922 and *Staphylococcus aureus* ATCC 6538. Testing was performed in 96-well microtitre plates (Becton Dickinson, New Jersey USA). To determine the MIC for test compounds, overnight cultures were diluted 1:100 and delivered to each test well (100 µL). Stock solutions of test compounds (100 µL) were added to the first column of test organism and mixed. Twofold serial dilutions were then carried out across the plate using a multi-channel pipette, changing the tips at each dilution step. The plates were then incubated for 48 h in a standard incubator at 37 °C. Growth was detected as turbidity (495 nm), relative to an uninoculated well using a microtitre plate reader. MICs were expressed as the lowest concentration of synthesised analogue at which growth did not occur, i.e., that which inhibited continued growth in the presence of the compound. Each MIC determination was carried out in quadruplicate. Negative (sterile broth) and positive (overnight culture with no added dentifrice) controls were also included.

MBCs were determined using the microtitre plates set-up for the MIC determinations. Aliquots (1 µL) taken from each well up to and including the MIC endpoint were transferred and spot-plated onto the appropriate agar. MBCs were expressed as the lowest concentration of synthesised analogue at which growth was not observed after 5 days of incubation, i.e., that which inactivated all bacteria in the original inoculum.

## Results and discussion

**Scheme 1:**
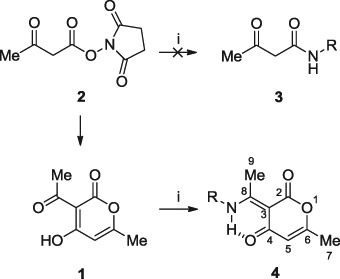
Synthesis of (*E*)-3-(1-aminoethylidene)-6-methyl-2*H*-pyran-2,4(3*H*)-diones **4** from **1** as opposed to intended acetoacetamide derivatives **3** from **2**. (i) RNH_2_, Et_3_N, DCM/DCE, r.t.−70 °C, 3–16 h

**Scheme 2:**
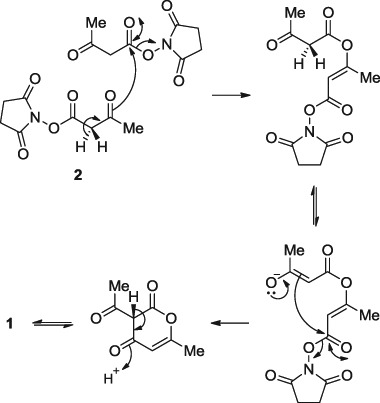
Proposed synthesis of **1** from **2** via Claisen condensation and intramolecular cyclisation

Reaction of commercially available **2** with a range of amines failed to give the expected acetoacetamides **3** based on NMR spectroscopy. This led to an analysis of the commercially available material **2** by ^1^H NMR spectroscopy to confirm its structure. However, the ^1^H NMR spectrum showed that **2** had cyclised into dehydroacetic acid **1** (Scheme 1), confirmed by comparing the ^1^H NMR and mass spectra with commercially available **1**. Compared to the original synthesis of **1** using ethyl acetoacetate and sodium carbonate (Arndt et al. 1940), the cyclisation of **2** into **1** represents a novel method for the preparation of **1**. We propose that two equivalents of **2** undergo Claisen condensation, followed by intramolecular nucleophilic substitution and subsequent loss of two equivalents of *N*-hydroxysuccinimide in support of the proposed reaction mechanism (Scheme 2).

Based on the unexpected synthesis of **1** and its known antibacterial activity, 12 enamine analogues of **1** were prepared by reacting equimolar equivalents of **1** with a selection of primary amines to afford the corresponding (*E*)-enaminopyran-2,4-diones (**4a**–**4l**, Scheme (1)). The ^1^H/^13^C NMR data obtained for novel analogues **4f–i** and **4k–l** along with their assignments are presented in Table 1. It has been previously shown that equimolar equivalents of **1** with ammonia or alkylamines favourably form (*E*)-enaminopyran-2,4-diones (Garratt 1963) while primary aromatic amines give the corresponding secondary enamines (Edwards et al. 1964). The characterisation data obtained for compounds **4a**–**e** and **4j** are in agreement with literature values (Garratt 1963; Edwards et al. 1964; Dias et al. 2009), whereas derivatives **4f–i** and **4k–l** are novel.

The antibacterial activities of **1** and analogues **4a**–**4l** were then examined on Gram-negative *Escherichia coli* and Gram-positive *Staphylococcus aureus* bacteria (Table 2). All synthesised derivatives except **4k–l** showed improved MIC values against *S. aureus* compared to **1**, while compounds **4a–f**, **4h** and **4k** demonstrated improved MIC values against *E. coli* when compared to **1**. The MBC of each derivative was also measured against *E. coli* and *S. aureus* and the results showed that derivatives **4a**–**4d**, **4f** and **4g** have greater bactericidal activities against both bacterial strains than **1**.

**Table 2:** Antibacterial activity of **1** and amino-substituted analogues **4a**–**4l** against one Gram-positive (*S. aureus*) and one Gram-negative bacterium (*E. coli*)

Compounds	MW	R	*E. coli*		*S. aureus*	
			MIC	MBC	MIC	MBC
**1**	168.15	—	0.42	1.69	0.84	1.69
**4a**	167.16	H	0.17	0.66	0.17	1.33
**4b**	181.19	CH_3_	0.08	0.15	0.3	0.6
**4c**	195.22	CH_2_CH_3_	0.38	1.51	0.38	0.38
**4d**	243.26	Ph	0.35	1.4	0.18	0.35
**4e**	257.29	CH_2_Ph	0.38	2.05 (0.9)	0.38	0.38
**4f**	271.32	CH_2_CH_2_Ph	0.3	1.2	0.3	1.2
**4g**	249.31	Cyclohexyl	>6.5	1.63	0.41	1.63
**4h**	205.21	CH_2_C≡CH	0.32	1.87(1)	0.64	2.58
**4i**	277.70	3-Cl-Ph	0.80	>1.60	0.40	1.60
**4j**	277.70	4-Cl-Ph	1.00	>2.00	0.50	>2.00
**4k**	311.26	3-CF_3_-Ph	0.32	>2.55	1.00	2.55
**4l**	299.37	4-^t^Bu-Ph	NA	NT	NA	NT

Data were determined by broth dilution endpoint (*n* = 4). Units are mg mL^−1^. Where data varied between replicates, standard deviations are given in parenthesis

*NA* not active, *NT* not tested

Structure–activity relationship (SAR) analysis revealed a number of interesting features. It was found that increasing the alkyl chain length negatively affected antibacterial activity (R = H > Me > Et, **4a**–**4c**) (Table 2). Substitution with a phenyl ring (**4d**) improved activity, but similar to alkyl derivatives, increasing the alkyl linker between the N atom and phenyl ring (derivatives **4e**–**4f**) generally decreases antibacterial activity. The presence of an unsaturated phenyl ring was essential as saturation of the ring to the cyclohexyl derivative (**4g**) significantly impacted on activity, particularly against *E. coli*. Given that compound **4d** was the most effective compound against *S. aureus*, we then designed and synthesised derivatives **4i–l** in order to investigate the effect of ring substitutions on antibacterial activity. Although none of the synthesised analogues were more potent than **4d**, it was interesting to note that electron-withdrawing substituents reduced antibacterial activity against both *E. coli* and *S. aureus*. In particular, the activities of derivatives **4i** (R = 3-Cl-Ph) and **4j** (R = 4-Cl-Ph) were very similar, indicating the requirement for the phenyl ring to be electron-rich. Derivative **4l** (R = 4-^t^Bu-Ph) was inactive against both *E. coli* and *S. aureus*, most likely due to steric reasons. Overall, **4b** was the best derivative identified in the series, with an MIC of 80 µg mL^−1^ and an MBC of 150 µg mL^−1^ against *E. coli* and an MIC of 300 µg mL^−1^ and an MBC of 600 µg mL^−1^ against *S. aureus*.

The ability of compounds **4b** and **4d** to inhibit and prevent the growth of both Gram-positive and Gram-negative bacteria at much lower concentrations than the lead compound **1** is promising. This cross-species antibacterial effect suggests a mode of action common among bacteria essential for its survival and growth. α-Pyrones have recently been identified as important endogenous signalling molecules in bacterial cell–cell communication (Brachmann et al. 2013) and it has been suggested that α-pyrones could have antagonist effects by disrupting quorum-like signalling in pyrone-producing bacteria, similar to the antimicrobial effects of synthetic analogues of *N*-acyl homoserine lactone (AHL) autoinducers on quorum sensing (Swem et al. 2009). The results of this study support the notion that some α-pyrone derivatives exhibit antibacterial properties and that the potential mechanism of action of pyrone derivatives **4b** and **4d** may involve interference with cell–cell bacterial communication. It is interesting to note that the α-pyrone signalling molecules described by Brachmann et al. had long fatty acid side chains and future analogues of **4** with these R groups may enhance their antibacterial activity. There are also reports that derivatives of **1** bind to and cleave DNA (Chitrapriya et al. 2008) and are suggested to target the essential bacterial enzyme DNA gyrase (Pal et al. 2014). Chemoproteomic studies using **4h** (R = propargyl) as a reagent for the ‘Click-chemistry’ method could be undertaken to elucidate the mechanism of action of these α-pyrone derivatives and potentially give rise to a new class of antibacterial agents.

## Conclusions

A novel synthesis of **1** is proposed based on the dimerisation of **2**. A number of (*E*)-enaminopyran-2,4-diones based on the structure of **1** were prepared and evaluated for antibacterial activity against Gram-positive and Gram-negative bacteria and demonstrate broad-spectrum activities. We identified **4b** (R = Me) and **4d** (R = Ph) as improved antibacterial agents that inhibited and prevented the growth of both *E. coli* and *S. aureus* to a greater extent than **1**. Derivative **4b** significantly inhibited the growth of *E. coli* compared to *S. aureus* while derivative **4d** had greater potency against *S. aureus* than *E. coli*. Further research is necessary to investigate their proposed mechanism of action as antagonists of bacterial communication as a potential avenue for antimicrobial drug development. Given the rise in antibiotic resistance, antibacterial agents based on the lead compounds **4b**/**4d** may be of use in the search for broad-spectrum antibiotics.

## Electronic supplementary material

Supplementary Material
