# Supplementary material for: Synthesis and antibacterial activities of enamine derivatives of dehydroacetic acid
Source: Med Chem Res. 2017 Nov 13;27(3):884–9. doi: 10.1007/s00044-017-2110-8 (PMC5835058; doi:10.1007/s00044-017-2110-8)
Supplement: Supplementary file 1 — Supplementary Material [file 44_2017_2110_MOESM1_ESM.docx]

**Electronic Supplementary Material**

**Synthesis and antibacterial activities of enamine derivatives of dehydroacetic acid**

**Alex G. Baldwin^1^ · Jonathan Bevan^1^ · David Brough^2^ · Ruth Ledder^1^ · Sally Freeman^1^**

✉ Sally Freeman

[sally.freeman@manchester.ac.uk](mailto:sally.freeman@manchester.ac.uk)

Alex G. Baldwin

[alex.baldwin@manchester.ac.uk](mailto:alex.baldwin@manchester.ac.uk)

Jonathan Bevan

[jonathan.bevan1@gmail.com](mailto:jonathan.bevan1@gmail.com)

David Brough

[david.brough@manchester.ac.uk](mailto:david.brough@manchester.ac.uk)

Ruth Ledder

[ruth.ledder@manchester.ac.uk](mailto:ruth.ledder@manchester.ac.uk)

^1^Division of Pharmacy & Optometry, School of Health Sciences, Faculty of Biology, Medicine and Heath, The University of Manchester, Stopford Building, Oxford Road, Manchester, M13 9PT, U.K.

^2^Division of Neuroscience and Experimental Psychology, School of Biological Sciences, Faculty of Biology, Medicine and Health, The University of Manchester, AV Hill Building, Oxford Road, Manchester, M13 9PT, U.K.

**Contents**

[Characterisation data for **4a-4e and 4j** S4](#_Toc496785083)

[^1^H NMR spectrum (CDCl_3_) of (*E*)-3-(1-aminoethylidene)-6-methyl-2*H*-pyran-2,4(3*H*)-dione (**4a**) S7](#_Toc496785084)

[^13^C NMR spectrum (CDCl_3_) of (*E*)-3-(1-aminoethylidene)-6-methyl-2*H*-pyran-2,4(3*H*)-dione (**4a**) S8](#_Toc496785085)

[HRMS(APCI^+^ TOF-MS) of (*E*)-3-(1-aminoethylidene)-6-methyl-2*H*-pyran-2,4(3*H*)-dione (**4a**) S9](#_Toc496785086)

[^1^H NMR spectrum (CDCl_3_) of (*E*)-6-methyl-3-(1-(methylamino)ethylidene)-2*H*-pyran-2,4(3*H*)-dione (**4b**) S10](#_Toc496785087)

[^13^C NMR spectrum (CDCl_3_) of (*E*)-6-methyl-3-(1-(methylamino)ethylidene)-2*H*-pyran-2,4(3*H*)-dione (**4b**) S11](#_Toc496785088)

[HRMS(ESI^+^) of (E)-6-methyl-3-(1-(methylamino)ethylidene)-2H-pyran-2,4(3H)-dione (**4b**) S12](#_Toc496785089)

[^1^H NMR spectrum (CDCl_3_) of (*E*)-3-(1-(ethylamino)ethylidene)-6-methyl-2*H*-pyran-2,4(3*H*)-dione (**4c**) S13](#_Toc496785090)

[^13^C NMR spectrum (CDCl_3_) of (*E*)-3-(1-(ethylamino)ethylidene)-6-methyl-2*H*-pyran-2,4(3*H*)-dione (**4c**) S14](#_Toc496785091)

[HRMS(APCI^+^ TOF-MS) of (*E*)-3-(1-(ethylamino)ethylidene)-6-methyl-2*H*-pyran-2,4(3*H*)-dione (**4c**) S15](#_Toc496785092)

[^1^H NMR spectrum (CDCl_3_) of (*E*)-6-methyl-3-(1-(phenylamino)ethylidene)-2*H*-pyran-2,4(3*H*)-dione (**4d**) S16](#_Toc496785093)

[^13^C NMR spectrum (CDCl_3_) of (*E*)-6-methyl-3-(1-(phenylamino)ethylidene)-2*H*-pyran-2,4(3*H*)-dione (**4d**) S17](#_Toc496785094)

[HRMS(ESI^+^ TOF-MS) of (*E*)-6-methyl-3-(1-(phenylamino)ethylidene)-2*H*-pyran-2,4(3*H*)-dione (**4d**) S18](#_Toc496785095)

[^1^H NMR spectrum (CDCl_3_) of (*E*)-3-(1-(benzylamino)ethylidene)-6-methyl-2*H*-pyran-2,4(3*H*)-dione (**4e**) S19](#_Toc496785096)

[^13^C NMR spectrum (CDCl_3_) of (*E*)-3-(1-(benzylamino)ethylidene)-6-methyl-2*H*-pyran-2,4(3*H*)-dione (**4e**) S20](#_Toc496785097)

[HRMS(APCI^+^ TOF-MS) of (*E*)-3-(1-(benzylamino)ethylidene)-6-methyl-2*H*-pyran-2,4(3*H*)-dione (**4e**) S21](#_Toc496785098)

[^1^H NMR spectrum (CDCl_3_) of (*E*)-6-methyl-3-(1-(phenethylamino)ethylidene)-2*H*-pyran-2,4(3*H*)-dione (**4f**) S22](#_Toc496785099)

[^13^C NMR spectrum (CDCl_3_) of (*E*)-6-methyl-3-(1-(phenethylamino)ethylidene)-2*H*-pyran-2,4(3*H*)-dione (**4f**) S23](#_Toc496785100)

[HRMS(APCI^+^ TOF-MS) of (*E*)-6-methyl-3-(1-(phenethylamino)ethylidene)-2*H*-pyran-2,4(3*H*)-dione (**4f**) S24](#_Toc496785101)

[^1^H NMR spectrum (CDCl_3_) of (*E*)-3-(1-(cyclohexylamino)ethylidene)-6-methyl-2*H*-pyran-2,4(3*H*)-dione (**4g**) S25](#_Toc496785102)

[^13^C NMR spectrum (CDCl_3_) of (*E*)-3-(1-(cyclohexylamino)ethylidene)-6-methyl-2*H*-pyran-2,4(3*H*)-dione (**4g**) S26](#_Toc496785103)

[HRMS(APCI^+^ TOF-MS) of (*E*)-3-(1-(cyclohexylamino)ethylidene)-6-methyl-2*H*-pyran-2,4(3*H*)-dione (**4g**) S27](#_Toc496785104)

[^1^H NMR spectrum (CDCl_3_) of (*E*)-6-methyl-3-(1-(prop-2-yn-1-ylamino)ethylidene)-2*H*-pyran-2,4(3*H*)-dione (**4h**) S28](#_Toc496785105)

[^13^C NMR spectrum (CDCl_3_) of (*E*)-6-methyl-3-(1-(prop-2-yn-1-ylamino)ethylidene)-2*H*-pyran-2,4(3*H*)-dione (**4h**) S29](#_Toc496785106)

[HRMS(APCI^+^ TOF-MS) of (*E*)-6-methyl-3-(1-(prop-2-yn-1-ylamino)ethylidene)-2*H*-pyran-2,4(3*H*)-dione (**4h**) S30](#_Toc496785107)

[^1^H NMR spectrum (CDCl_3_) of (*E*)-3-(1-(3-chlorophenylamino)ethylidene)-6-methyl-2*H*-pyran-2,4(3*H*)-dione (**4i**) S31](#_Toc496785108)

[^13^C NMR spectrum (CDCl_3_) of (*E*)-3-(1-(3-chlorophenylamino)ethylidene)-6-methyl-2*H*-pyran-2,4(3*H*)-dione (**4i**) S32](#_Toc496785109)

[HRMS(ESI^+^) of (*E*)-3-(1-(3-chlorophenylamino)ethylidene)-6-methyl-2*H*-pyran-2,4(3*H*)-dione (**4i**).... S33](#_Toc496785110)

[^1^H NMR spectrum (CDCl_3_) of (*E*)-3-(1-(4-chlorophenylamino)ethylidene)-6-methyl-2*H*-pyran-2,4(3*H*)-dione (**4j**) S34](#_Toc496785111)

[^13^C NMR spectrum (CDCl_3_) of (*E*)-3-(1-(4-chlorophenylamino)ethylidene)-6-methyl-2*H*-pyran-2,4(3*H*)-dione (**4j**) S35](#_Toc496785112)

[HRMS(ESI^+^) of (*E*)-3-(1-(4-chlorophenylamino)ethylidene)-6-methyl-2*H*-pyran-2,4(3*H*)-dione (**4j**) .S36](#_Toc496785113)

[^1^H NMR spectrum (CDCl_3_) of (*E*)-6-methyl-3-(1-((3-(trifluoromethyl)phenyl)amino)ethylidene)-2*H*-pyran-2,4(3*H*)-dione (**4k**) S37](#_Toc496785114)

[^13^C NMR spectrum (CDCl_3_) of (*E*)-6-methyl-3-(1-((3-(trifluoromethyl)phenyl)amino)ethylidene)-2*H*-pyran-2,4(3*H*)-dione (**4k**) S38](#_Toc496785115)

[HRMS(ESI^+^) of (*E*)-6-methyl-3-(1-((3-(trifluoromethyl)phenyl)amino)ethylidene)-2*H*-pyran-2,4(3*H*)-dione (**4k**) S39](#_Toc496785116)

[^1^H NMR spectrum (CDCl_3_) of (*E*)-3-(1-((4-(*tert*-butyl)phenyl)amino)ethylidene)-6-methyl-2*H*-pyran-2,4(3*H*)-dione (**4l**) S40](#_Toc496785117)

[^13^C NMR spectrum (CDCl_3_) of (*E*)-3-(1-((4-(*tert*-butyl)phenyl)amino)ethylidene)-6-methyl-2*H*-pyran-2,4(3*H*)-dione (**4l**) S41](#_Toc496785118)

[HRMS(ESI^+^) of (*E*)-3-(1-((4-(*tert*-butyl)phenyl)amino)ethylidene)-6-methyl-2*H*-pyran-2,4(3*H*)-dione (**4l**) S42](#_Toc496785119)

# **Characterisation data for 4a-4e and 4j**

**(*E*)-3-(1-Aminoethylidene)-6-methyl-2*H*-pyran-2,4(3*H*)-dione (4a)**. White solid. 100% EtOAc. Yield: 30%. mp 210-211^o^C (lit. 210-211^o^C ([Edwards et al., 1964](#_ENREF_6))); IR (neat): ν_max_ 3234, 1712, 1682, 1568, 1463, 1353, 1000 cm^-1^; ^1^H NMR (CDCl_3_, 300 MHz): δ = 12.87 (1H, br s, NH), 6.63 (1H, br s, NH), 5.70 (1H, s, H5), 2.64 (3H, s, CH­_3_), 2.13 (3H, s, CH_3_); ^13^C NMR (CDCl_3_, 75 MHz): δ = 185.0 (C8), 177.0 (C4), 163.5 (C2), 163.3 (C6), 107.5 (C5), 96.9 (C3), 26.1 (C9), 19.9 (C7); MS(ESI^+^) (*m*/*z*, %): 168.1 [M+H, 13]^+^, 190.1 [M+Na, 100]^+^; HRMS(APCI^+^ TOF-MS) (*m*/*z*): [M+H]^+^ calcd for C_8_H_10_NO_3_, 168.0661, found: 168.0668, error: 4.2 ppm.

**(*E*)-6-Methyl-3-(1-(methylamino)ethylidene)-2*H*-pyran-2,4(3*H*)-dione (4b)**. White solid. EtOAc:*n*-hexane (1:1). Yield: 61%. mp 123-124^o^C (lit. 126-127^o^C ([Garratt, 1963](#_ENREF_8))); IR (neat): ν_max_ 1686, 1654, 1575, 1466, 1340, 997 cm^-1^; ^1^H NMR (CDCl_3_, 300 MHz): δ = 14.06 (1H, br s, NH), 5.69 (1H, s, H5), 3.16 (3H, d, *J* = 5.1 Hz, H1’), 2.64 (3H, s, CH_3_), 2.12 (3H, s, CH_3_); ^13^C NMR (CDCl_3_, 75 MHz): δ = 184.7 (C8), 177.0 (C4), 163.8 (C2), 162.6 (C6), 107.4 (C5), 96.7 (C3), 30.5 (C1’), 19.8 (C9), 17.8 (C7); MS(ESI^+^) (*m*/*z*): 182.0 [M+H, 41]^+^, 204.0 [M+Na, 100]^+^; HRMS(ESI^+^) (*m*/*z*): [M+H]^+^ calcd. for C_9_H_12_NO_3_, 182.0812, found: 182.0811, error: 0.5 ppm.

**(*E*)-3-(1-(Ethylamino)ethylidene)-6-methyl-2*H*-pyran-2,4(3*H*)-dione (4c)**. White solid. EtOAc:*n*-hexane (2:3). Yield: 96%. mp 90-91^o^C (lit. 87-88^o^C ([Garratt, 1963](#_ENREF_8))); IR (neat): ν_max_ 1683, 1653, 1571, 1472, 1360, 1329, 997 cm^-1^; ^1^H NMR (CDCl_3_, 300 MHz): δ = 14.11 (1H, br s, NH), 5.68 (1H, s, H5), 3.52 (2H, dq, *J* = 6.6 Hz & 6.6 Hz, H1’), 2.63 (3H, s, CH_3_), 2.12 (3H, s, CH_3_), 1.37 (3H, t, *J* = 7.4 Hz, H2’); ^13^C NMR (CDCl_3_, 75 MHz): δ = 184.7 (C8), 175.7 (C4), 163.9 (C2), 162.6 (C6), 107.4 (C5), 96.4 (C3), 39.0 (C1’), 19.8 (C9), 18.1 (C7), 14.4 (C2’); MS(ESI^+^) (*m*/*z*): 196.0 [M+H, 100]^+^, 218.0 [M+Na, 56]^+^; HRMS(APCI^+^ TOF-MS) (*m*/*z*): [M+H]^+^ calcd. for C_10_H_14_NO_3_, 196.0974, found: 196.0969, error: 2.5 ppm.

**(*E*)-6-Methyl-3-(1-(phenylamino)ethylidene)-2*H*-pyran-2,4(3*H*)-dione (4d)**. The reaction was refluxed in DCE instead of DCM at room temperature. Cream solid. EtOAc:*n*‑hexane (3:7). Yield: 42%. mp 121-122^o^C (lit. 127-128^o^C ([Dias et al., 2009](#_ENREF_5))); IR (neat): ν_max_ 1690, 1654, 1556, 1472, 1367, 1000 cm^-1^; ^1^H NMR (CDCl_3_, 400 MHz): δ = 15.79 (1H, br s, NH), 7.46 (2H, t, *J* = 7.4 Hz, H3’ and H5’), 7.38 (1H, t, *J* = 7.2 Hz, H4’), 7.18 (2H, d, *J*= 7.6 Hz, H2’ and H6’), 5.76 (1H, s, H5), 2.59 (3H, s, CH_3_), 2.16 (3H, s, CH_3_); ^13^C NMR (CDCl_3_, 100 MHz): δ = 185.1 (C8), 175.5 (C4), 163.63 (C2), 163.56 (C6), 136.5 (C1’), 129.7 (C2’ and C6’), 128.2 (C4’), 125.7 (C3’ and C5’), 107.3 (C5), 97.5 (C3), 20.5 (C9), 20.1 (C7); MS(ESI^+^) (*m*/*z*): 244.0 [M+H, 100]^+^, 266.0 [M+Na, 30]^+^; HRMS(ESI^+^ TOF‑MS) (*m*/*z*): [M+H]^+^ calcd. for C_14_H_13_NO_3_Na, 266.0793, found: 266.0789, error: 1.5 ppm.

**(*E*)-3-(1-(Benzylamino)ethylidene)-6-methyl-2*H*-pyran-2,4(3*H*)-dione (4e)**. Cream solid. EtOAc:*n*-hexane (1:3). Yield: 83%; mp 76-78^o^C (lit. 79-81^o^C ([Dias et al., 2009](#_ENREF_5))); IR (neat): ν_max_ 1694, 1654, 1567, 1472, 1357, 1324, 997 cm^-1^; ^1^H NMR (CDCl_3_, 300 MHz): δ = 14.47 (1H, br s, NH), 7.16-7.37 (5H, m, Ph), 5.62 (1H, s, H5), 4.62 (2H, d, *J*= 5.4 Hz, H1’), 2.60 (3H, s, CH_3_), 2.06 (3H, s, CH_3_); ^13^C NMR (CDCl_3_, 75 MHz): δ = 184.8 (C8), 176.4 (C4), 163.7 (C2), 162.8 (C6), 135.1 (C2’), 129.2 (C4’ and C6’), 128.3 (C5’), 127.1 (C3’ and C7’), 107.4 (C5), 96.9 (C3), 47.9 (C1’), 19.8 (C9), 18.3 (C7); MS(ESI^+^) (*m*/*z*): 258.1 [M+H, 100]^+^, 280.1 [M+Na, 44]^+^; HRMS(APCI^+^ TOF-MS) (*m*/*z*): [M+H]^+^ calcd. for C_15_H_16_NO_3_, 258.1130, found: 258.1127, error: 1.2 ppm.

**(*E*)-3-(1-(4-Chlorophenylamino)ethylidene)-6-methyl-2*H*-pyran-2,4(3*H*)-dione (4j)**. The reaction was refluxed in DCE instead of DCM at room temperature. Yellow solid. EtOAc:*n*-hexane (1:4). Yield: 28%. mp 140-141^o^C (lit. 138^o^C ([Edwards et al., 1964](#_ENREF_6))); ^1^H NMR (CDCl_3_, 300 MHz): δ = 7.43 (2H, d, *J* = 8.4 Hz, H2’ and H6’), 7.12 (2H, d, *J* = 8.4 Hz, H3’ and H5’), 5.76 (1H, s, H5), 2.58 (3H, s, CH_3_), 2.17 (3H, s, CH_3_); ^13^C NMR (CDCl_3­,_ 75 MHz): δ = 185.2 (C8), 175.6 (C4), 163.8 (C2), 163.4 (C6), 135.1 (C1’), 134.2 (C4’), 130.0 (C3’ and C5’), 127.1 (C2’ and C6’), 107.2 (C5), 97.7 (C3), 20.4 (C9), 20.1 (C7); MS(ESI^+^) (*m*/*z*): 278.1 [M+H, ^35^Cl, 100]^+^, 280.1 [M+H, ^37^Cl, 60]^+^, 300.1 [M+Na, ^35^Cl, 14]^+^, 302.1 [M+Na, ^37^Cl, 5]^+^; HRMS(ESI^+^) (*m*/*z*): [M+H]^+^ calcd. for C_14_H_13_^35^ClNO_3_, 278.0578, found: 278.0578, error: 0.0 ppm.


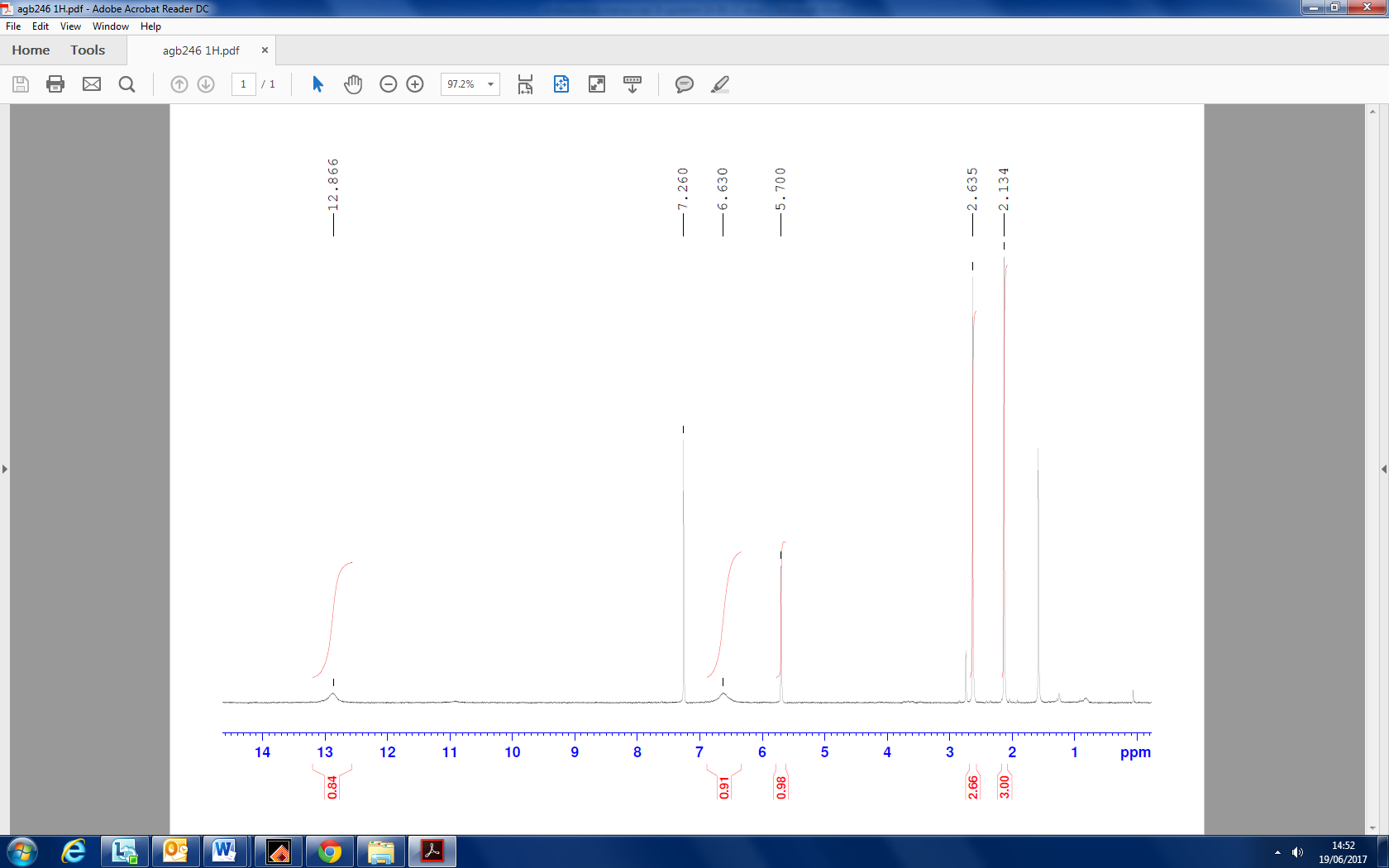

7

9

H_2_O

CHCl_3_

5

NH

NH

# ^1^H NMR spectrum (CDCl_3_) of (*E*)-3-(1-aminoethylidene)-6-methyl-2*H*-pyran-2,4(3*H*)-dione (**4a**)


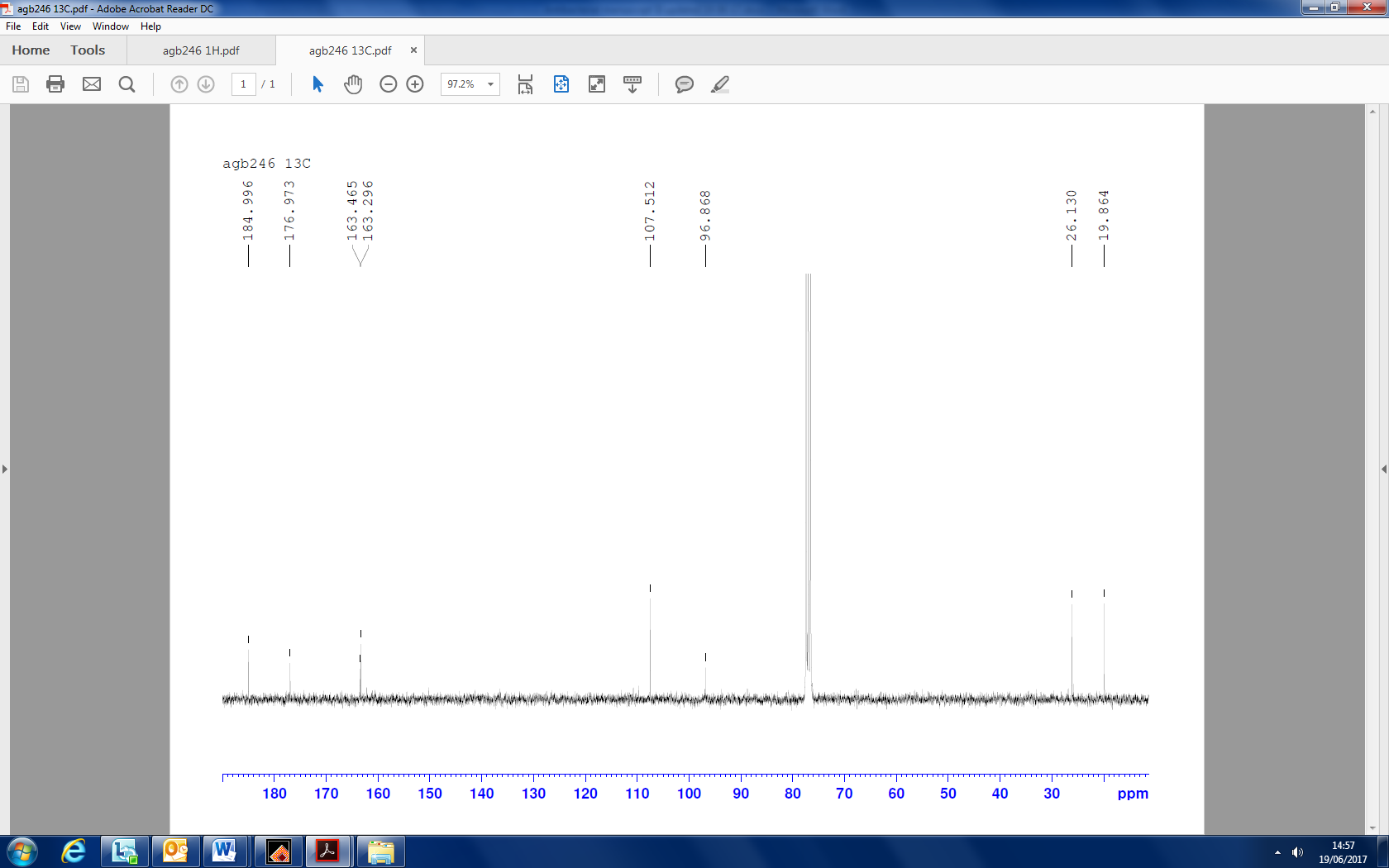


CHCl_3_

7

9

5

#

6

8

3

2

4

# ^13^C NMR spectrum (CDCl_3_) of (*E*)-3-(1-aminoethylidene)-6-methyl-2*H*-pyran-2,4(3*H*)-dione (**4a**)


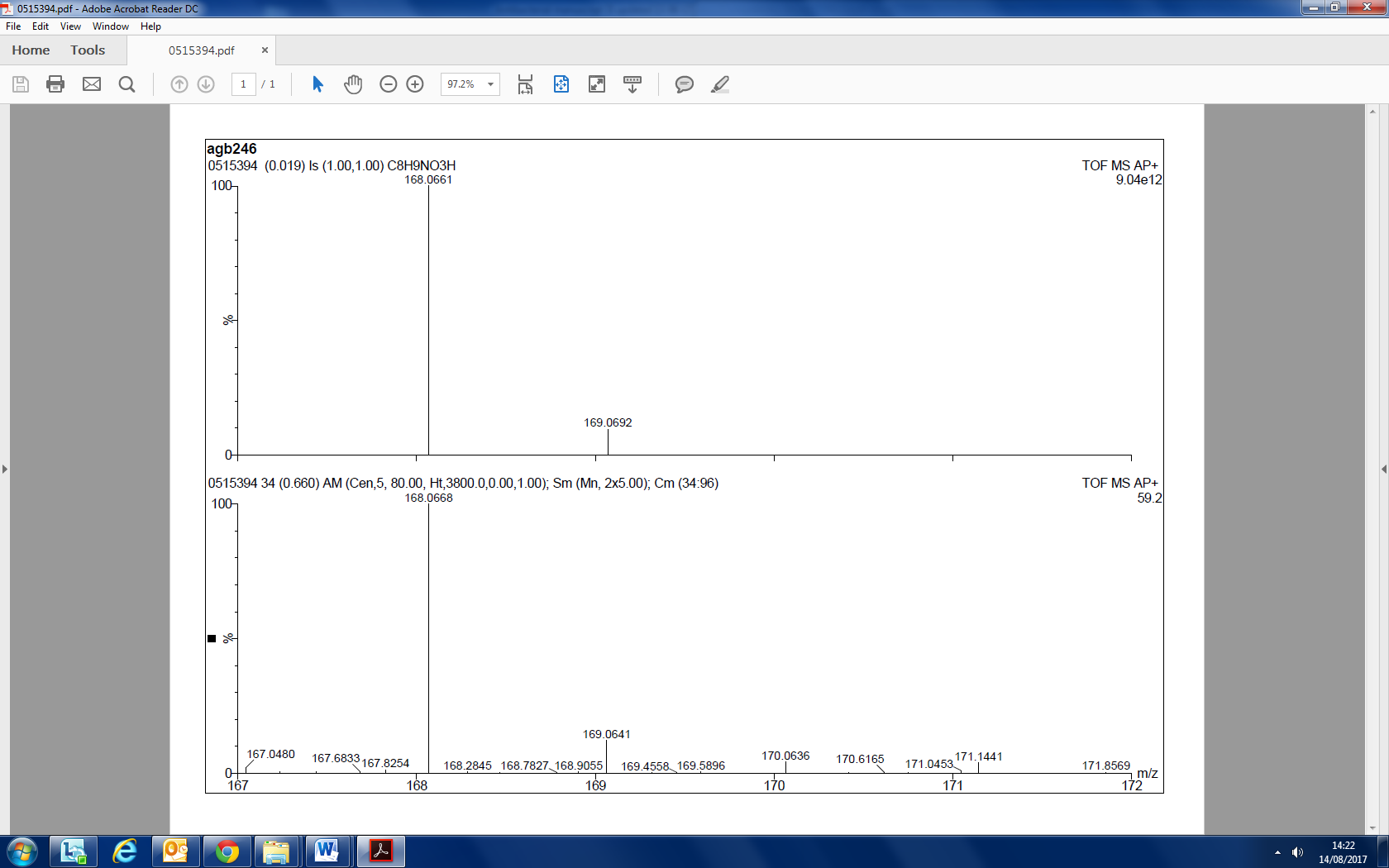


# HRMS(APCI^+^ TOF-MS) of (*E*)-3-(1-aminoethylidene)-6-methyl-2*H*-pyran-2,4(3*H*)-dione (**4a**)


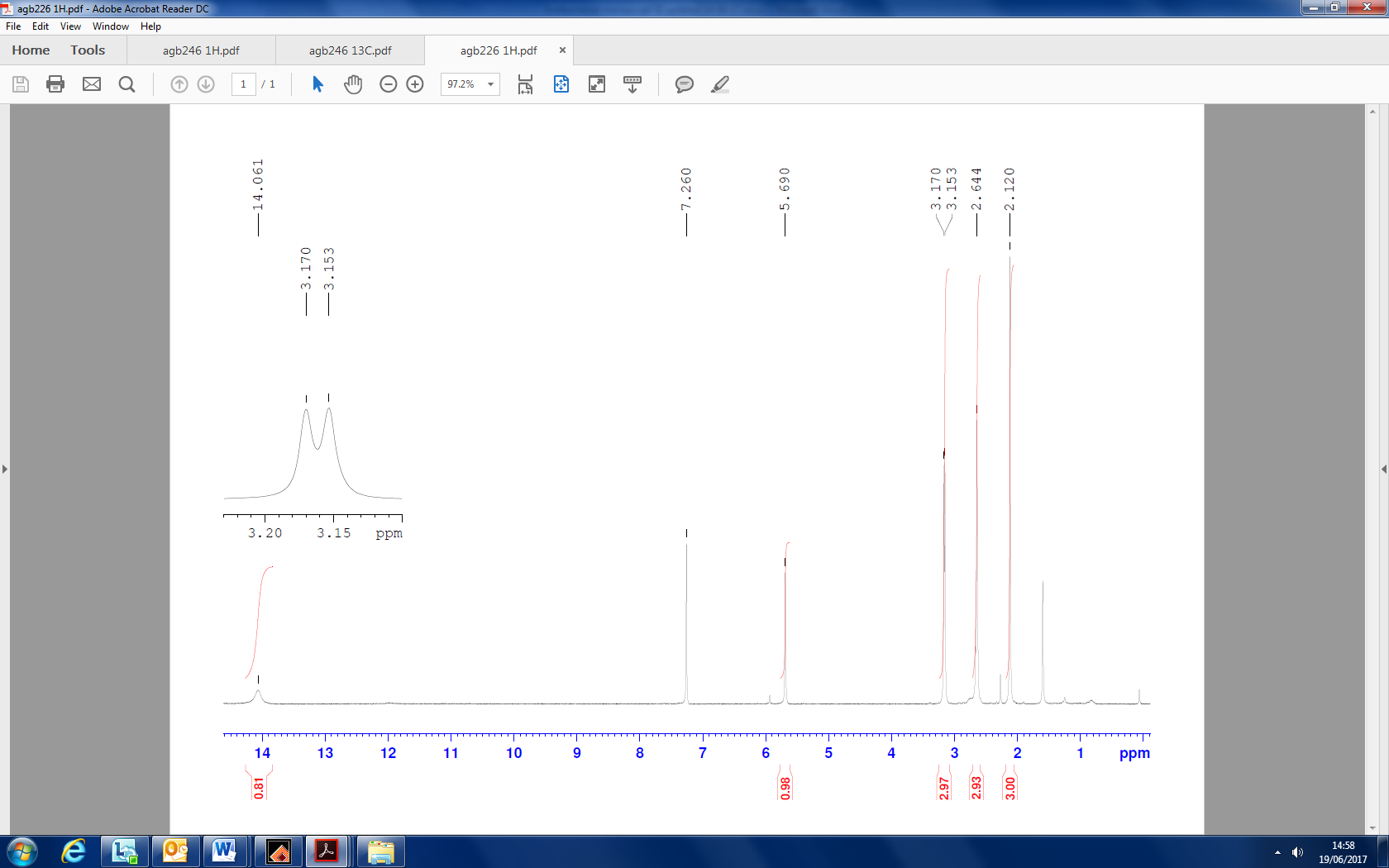

7

9

1’

CHCl_3_

H_2_O

5

NH

# ^1^H NMR spectrum (CDCl_3_) of (*E*)-6-methyl-3-(1-(methylamino)ethylidene)-2*H*-pyran-2,4(3*H*)-dione (**4b**)


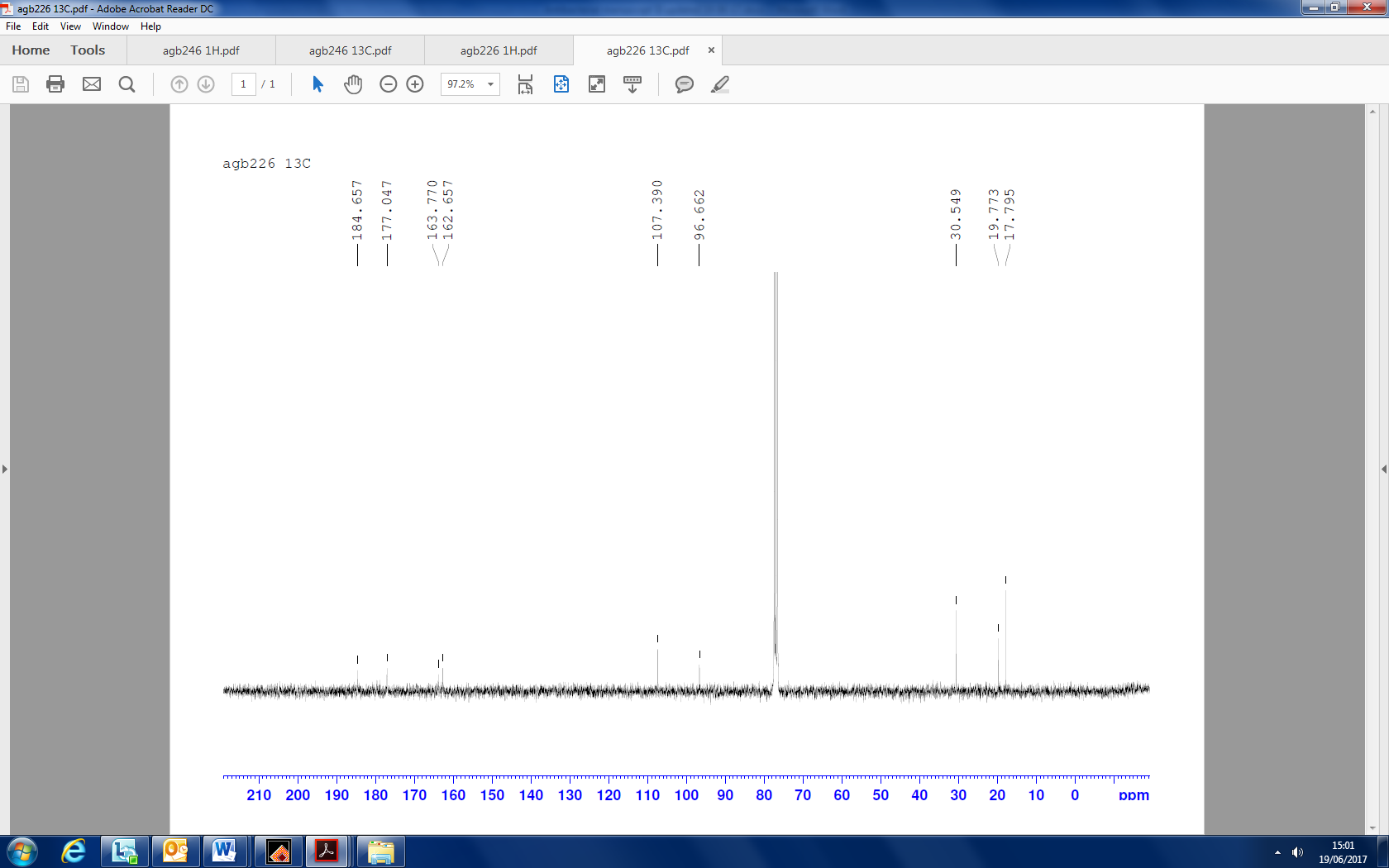

CHCl_3_

7

1’

9

3

5

6

4

8

2

# ^13^C NMR spectrum (CDCl_3_) of (*E*)-6-methyl-3-(1-(methylamino)ethylidene)-2*H*-pyran-2,4(3*H*)-dione (**4b**)

HRMS(ESI^+^) of (E)-6-methyl-3-(1-(methylamino)ethylidene)-2H-pyran-2,4(3H)-dione (**4b**)

^
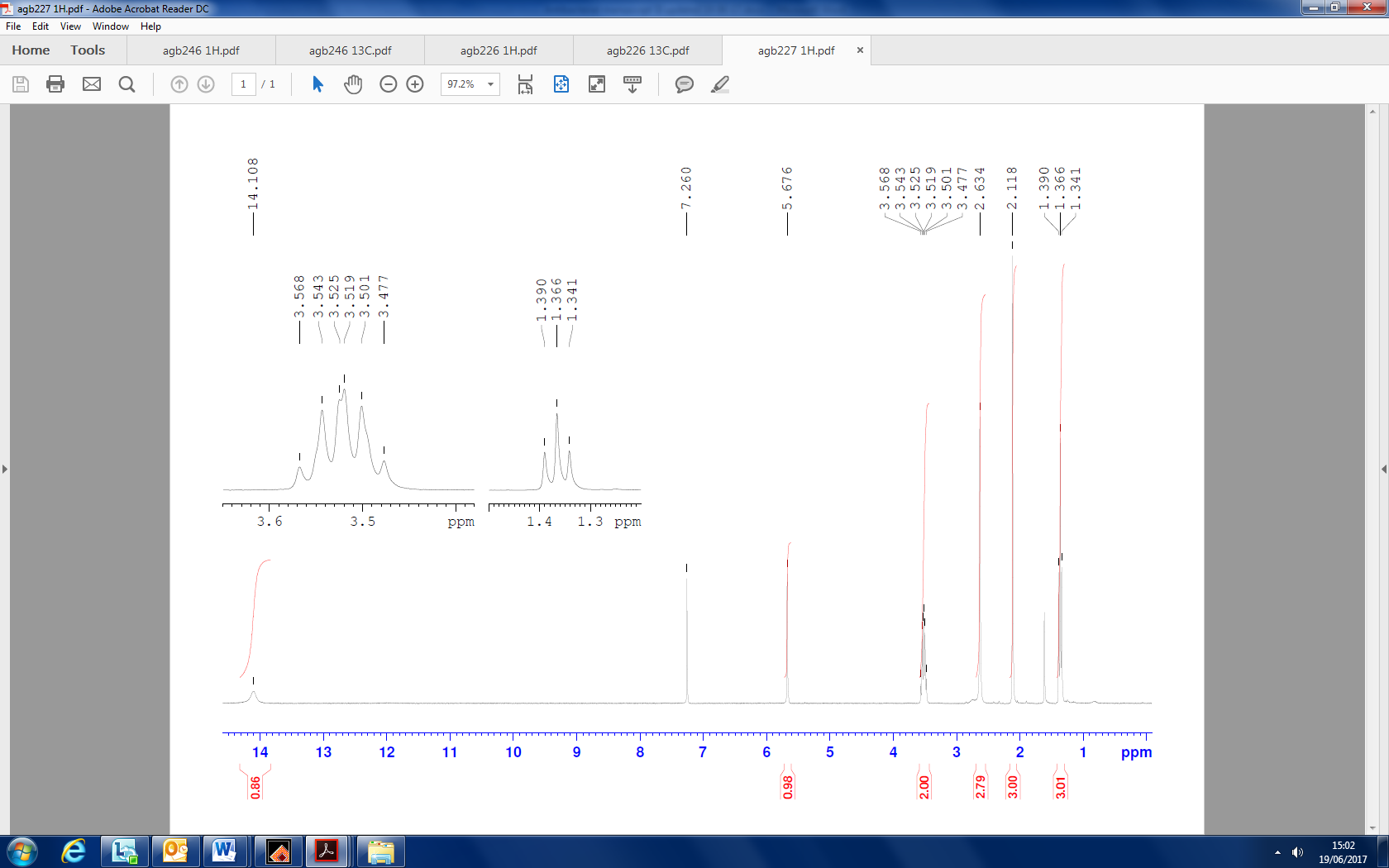
^

7

9

2’

1’

5

H_2_O

CHCl_3_

NH

# ^1^H NMR spectrum (CDCl_3_) of (*E*)-3-(1-(ethylamino)ethylidene)-6-methyl-2*H*-pyran-2,4(3*H*)-dione (**4c**)


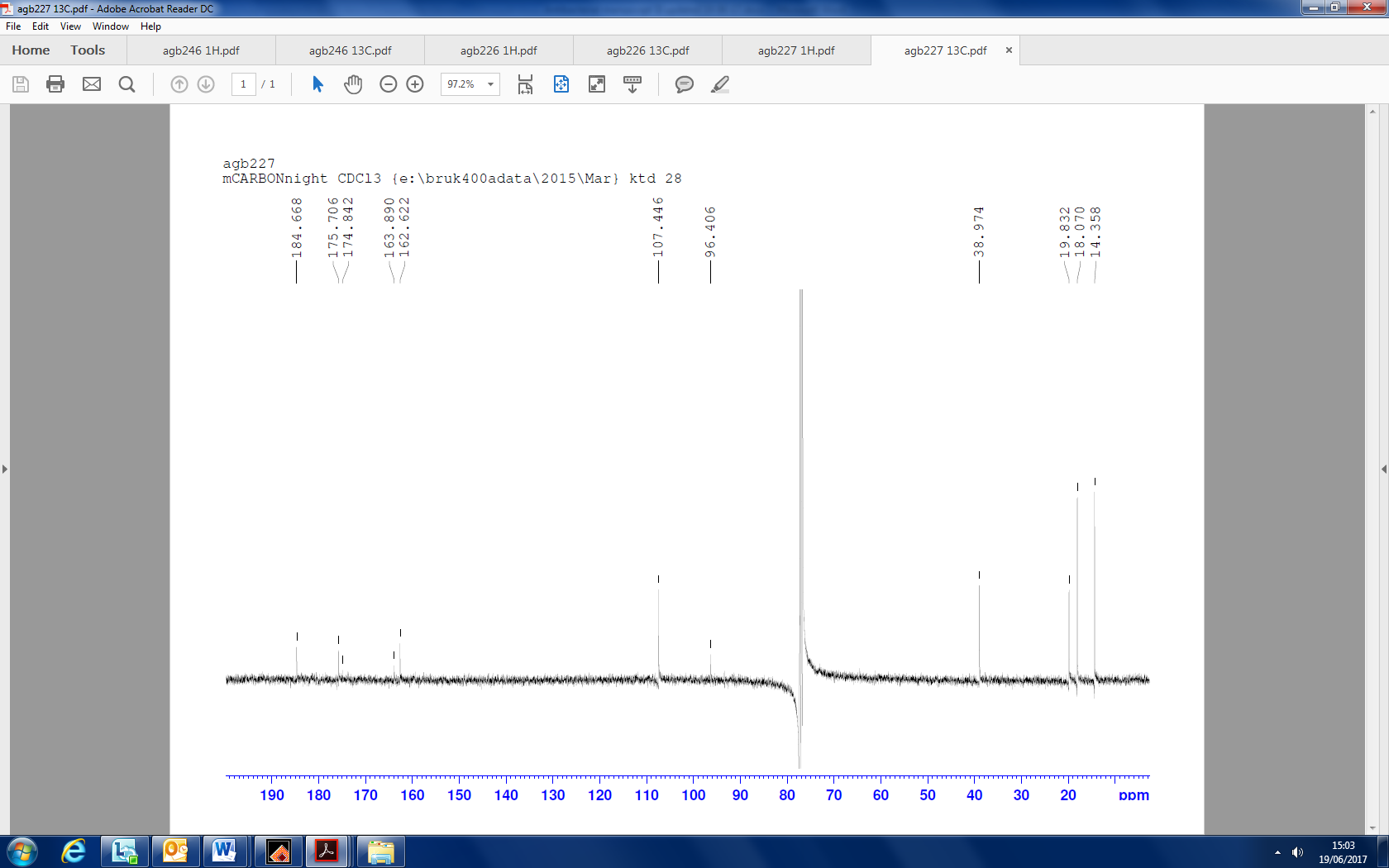

CHCl_3_

2’

7

9

1’

5

3

6

4

8

2

# ^13^C NMR spectrum (CDCl_3_) of (*E*)-3-(1-(ethylamino)ethylidene)-6-methyl-2*H*-pyran-2,4(3*H*)-dione (**4c**)


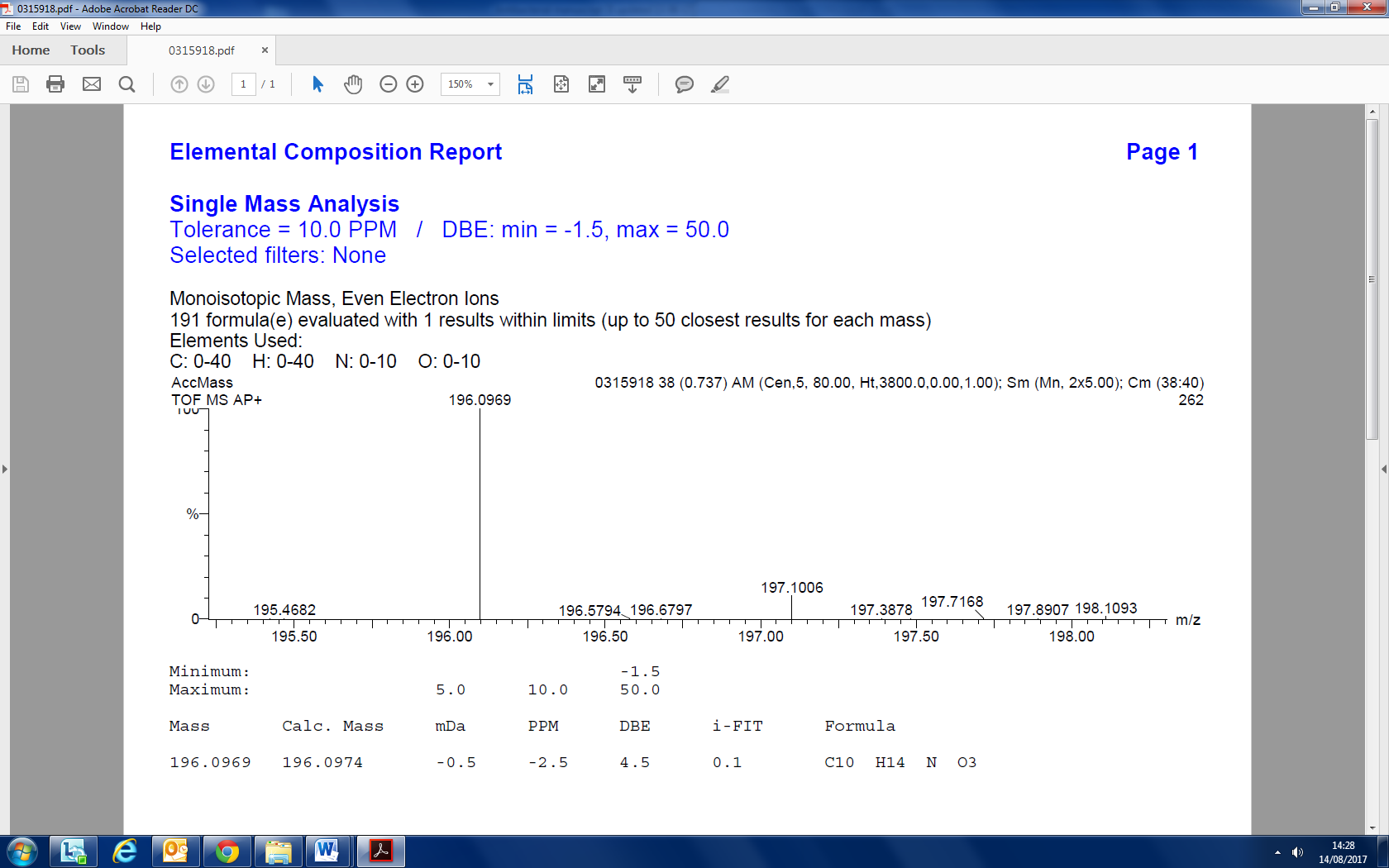


# HRMS(APCI^+^ TOF-MS) of (*E*)-3-(1-(ethylamino)ethylidene)-6-methyl-2*H*-pyran-2,4(3*H*)-dione (**4c**)

^
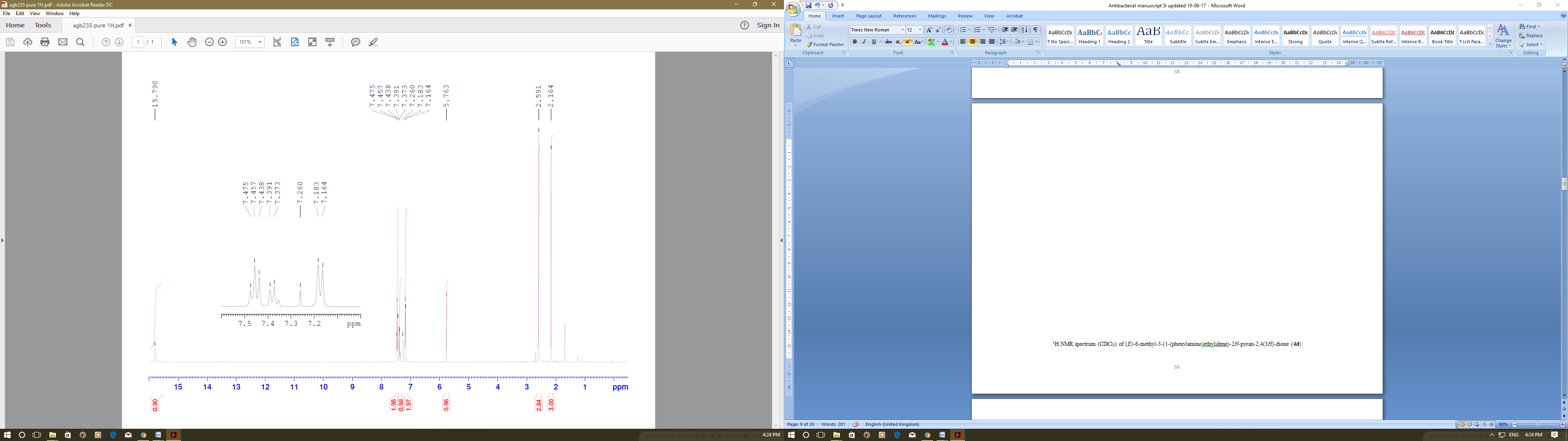
^

9

7

CHCl_3_

5

2’/6’

4’

3’/5’

NH

H_2_O

# ^1^H NMR spectrum (CDCl_3_) of (*E*)-6-methyl-3-(1-(phenylamino)ethylidene)-2*H*-pyran-2,4(3*H*)-dione (**4d**)


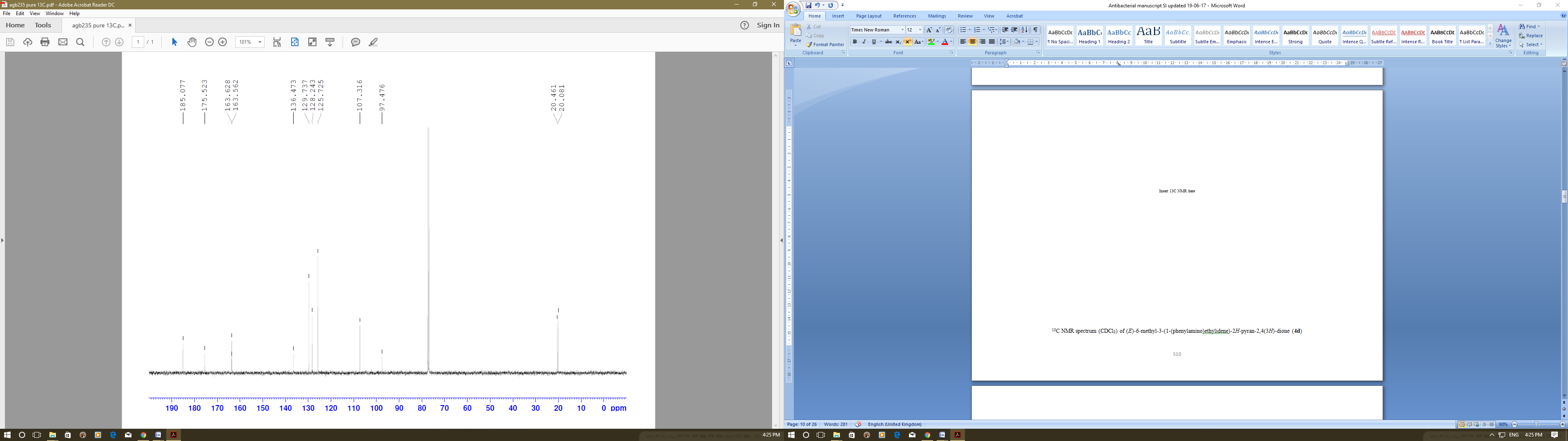


CHCl_3_

3’/5’

2’/6’

5

7

9

4’

3

1’

6

4

8

2

# ^13^C NMR spectrum (CDCl_3_) of (*E*)-6-methyl-3-(1-(phenylamino)ethylidene)-2*H*-pyran-2,4(3*H*)-dione (**4d**)


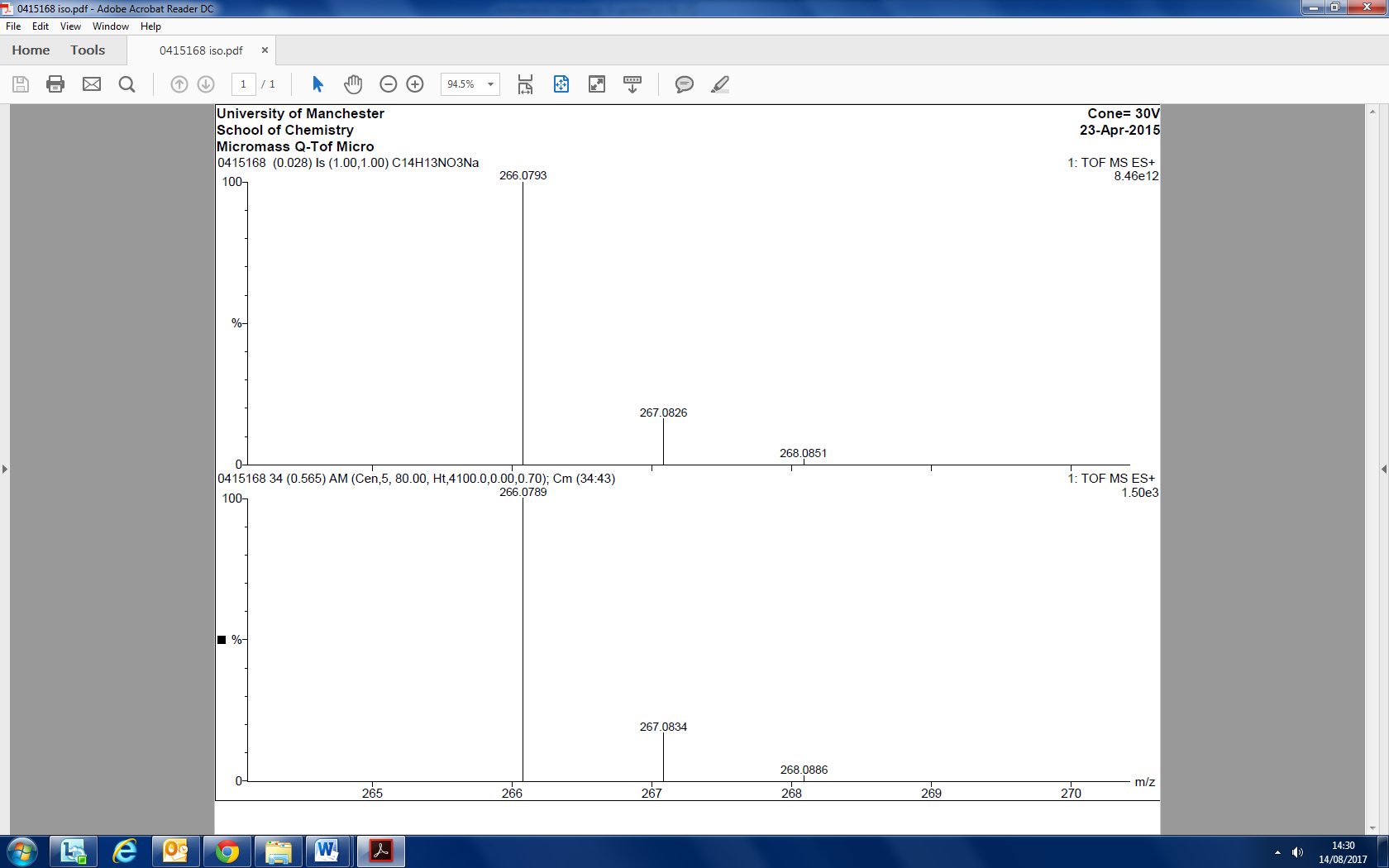


# HRMS(ESI^+^ TOF-MS) of (*E*)-6-methyl-3-(1-(phenylamino)ethylidene)-2*H*-pyran-2,4(3*H*)-dione (**4d**)

^
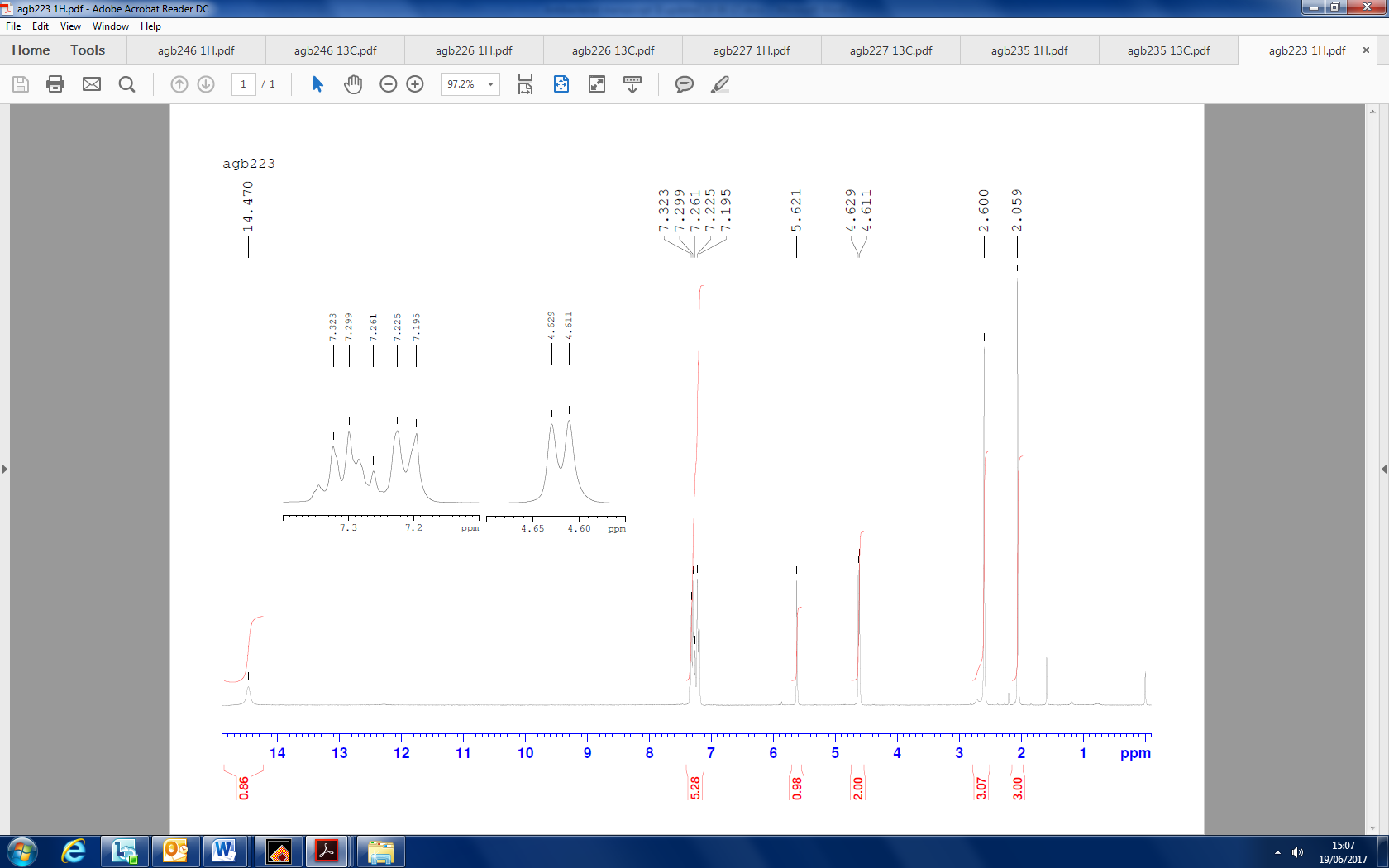
^

7

9

^
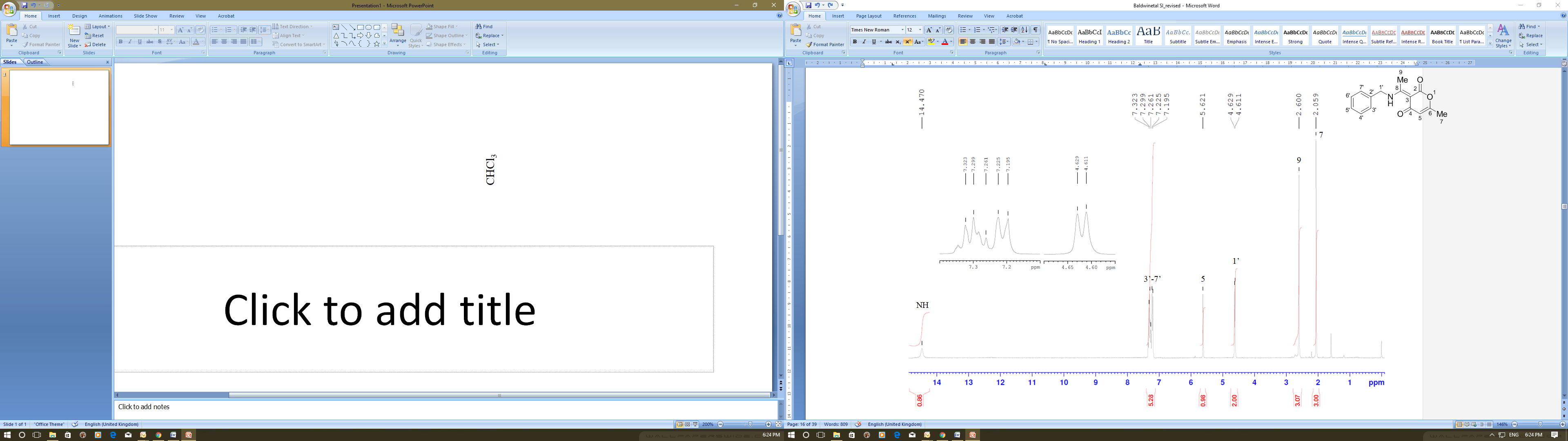
^

1’

5

3’-7’

NH

H_2_O

# ^1^H NMR spectrum (CDCl_3_) of (*E*)-3-(1-(benzylamino)ethylidene)-6-methyl-2*H*-pyran-2,4(3*H*)-dione (**4e**)


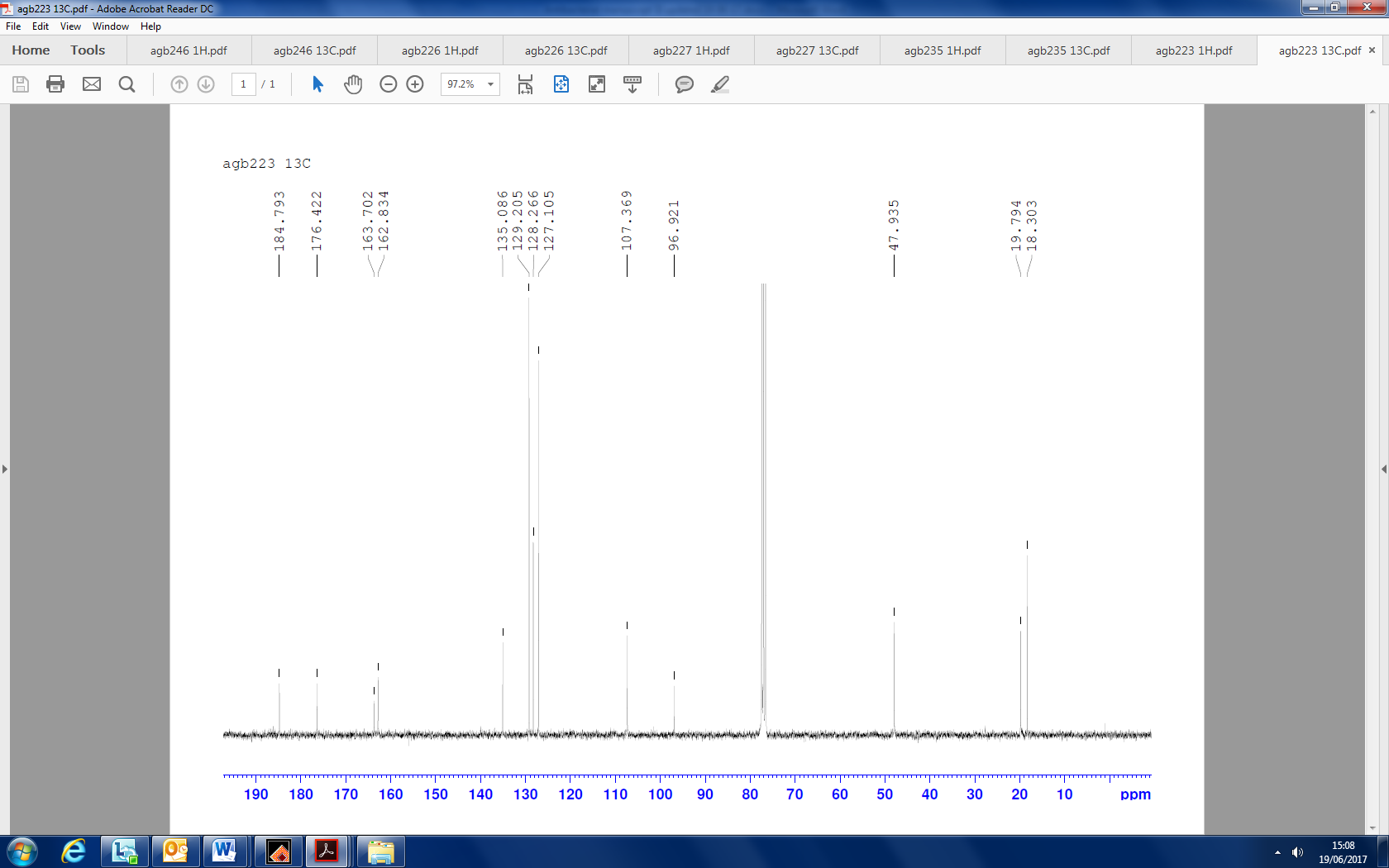


CHCl_3_

4’/6’

3’/7’

5’

7

1’

9

5

2’

3

2

6

4

8

# ^13^C NMR spectrum (CDCl_3_) of (*E*)-3-(1-(benzylamino)ethylidene)-6-methyl-2*H*-pyran-2,4(3*H*)-dione (**4e**)


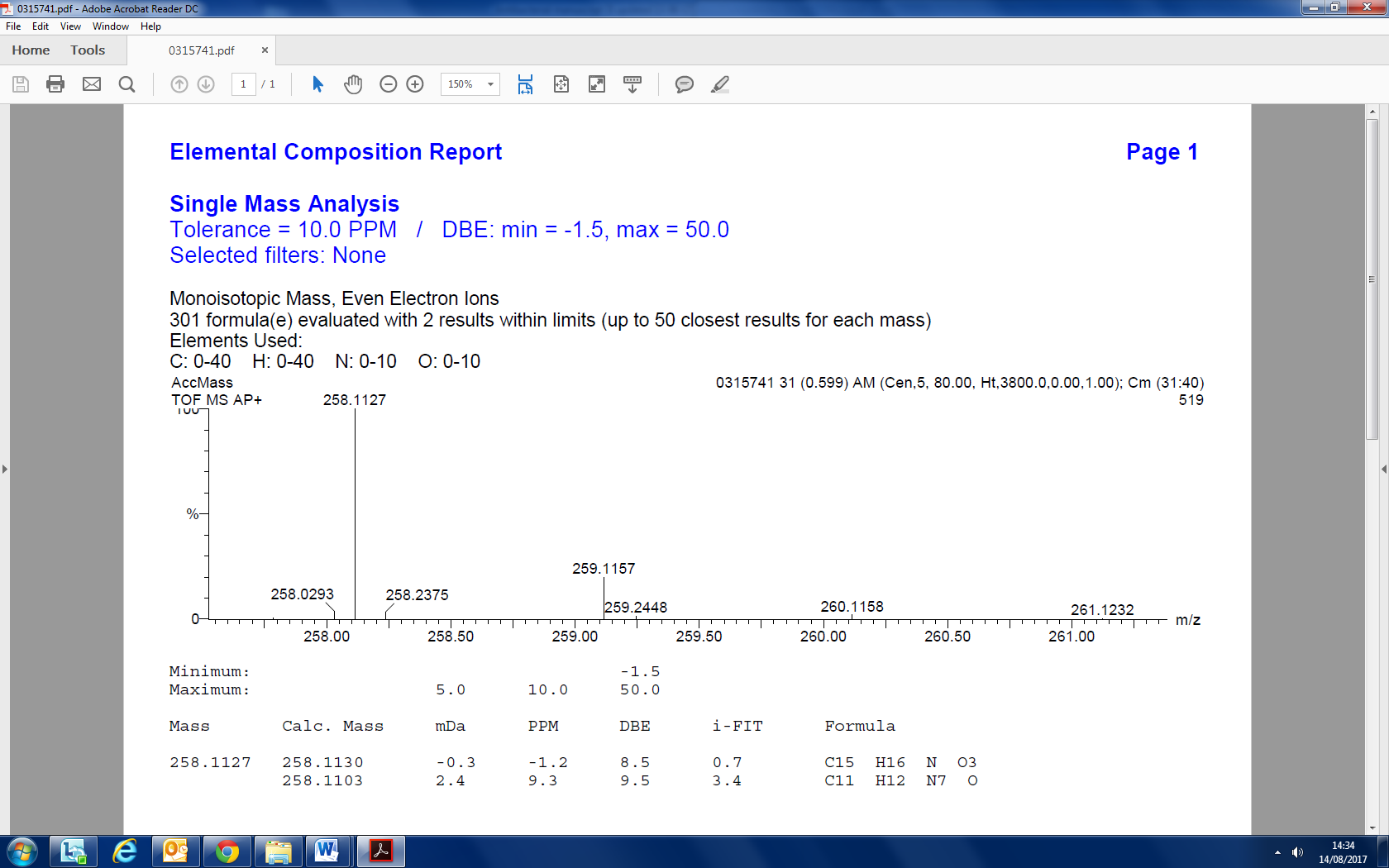


# HRMS(APCI^+^ TOF-MS) of (*E*)-3-(1-(benzylamino)ethylidene)-6-methyl-2*H*-pyran-2,4(3*H*)-dione (**4e**)


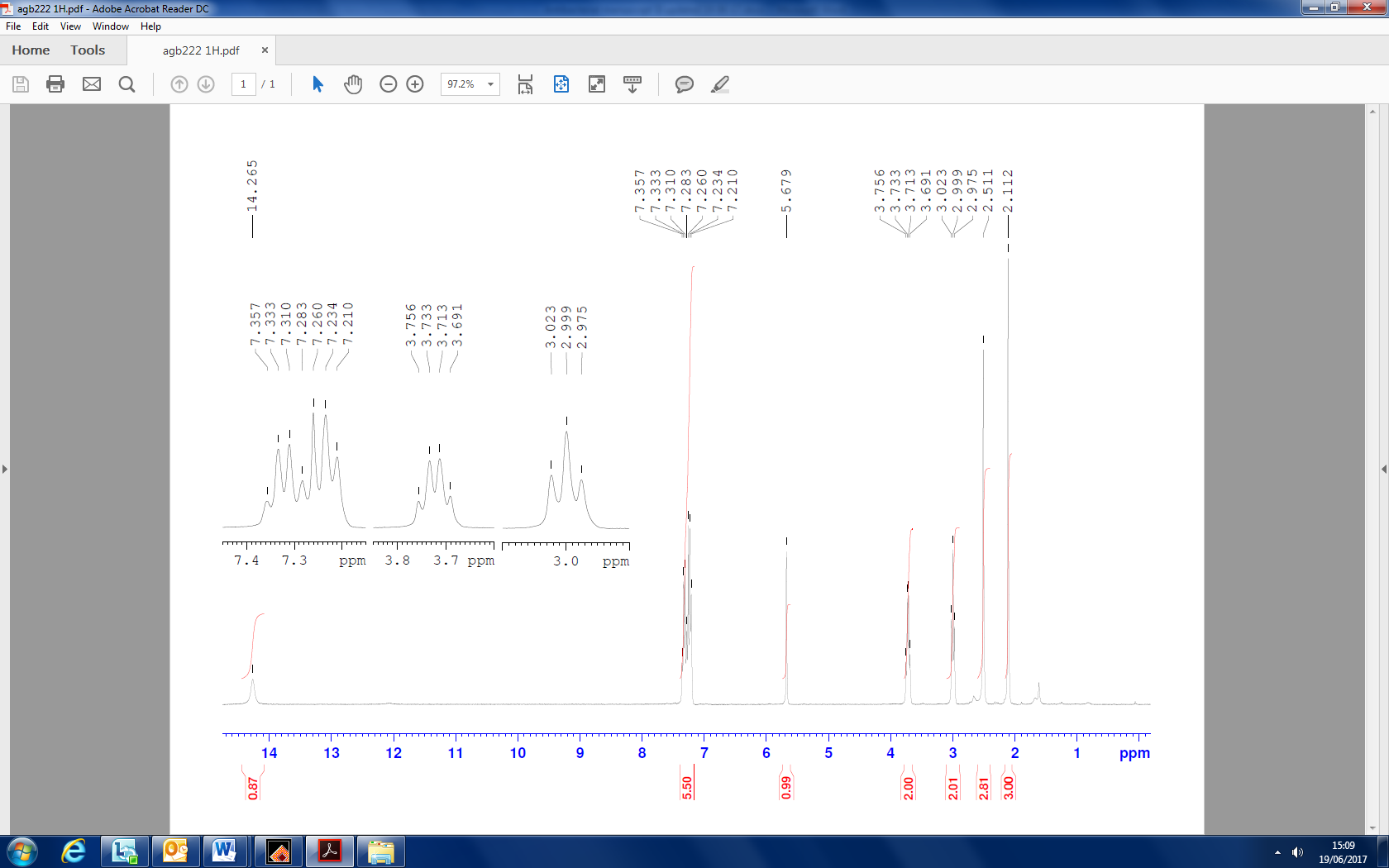

7

9

2’

1’

5

4’-8’

NH

# ^1^H NMR spectrum (CDCl_3_) of (*E*)-6-methyl-3-(1-(phenethylamino)ethylidene)-2*H*-pyran-2,4(3*H*)-dione (**4f**)


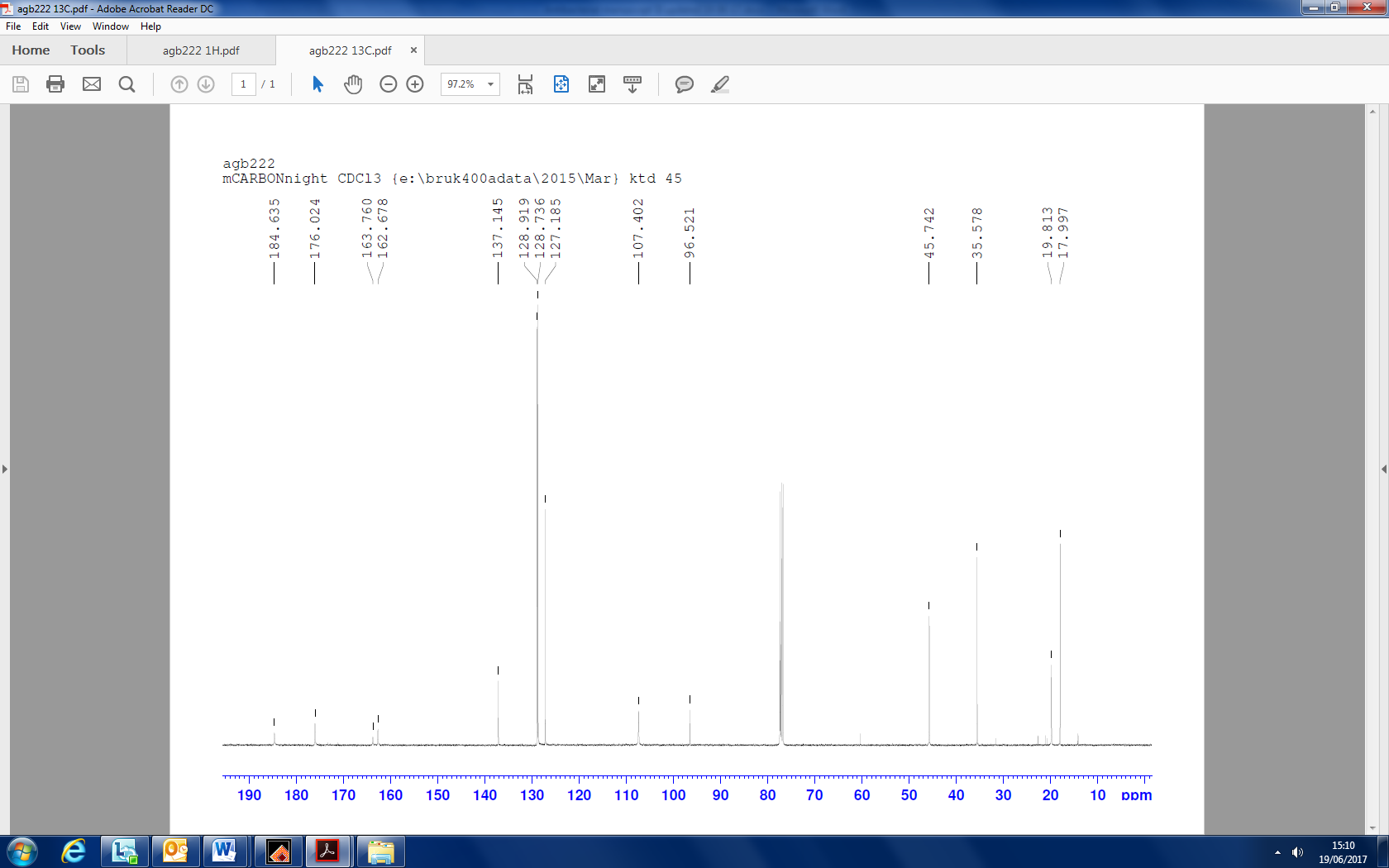

4’/8’

5’/7’

CHCl_3_

6’

7

2’

1’

9

3’

3

5

6

4

2

8

# ^13^C NMR spectrum (CDCl_3_) of (*E*)-6-methyl-3-(1-(phenethylamino)ethylidene)-2*H*-pyran-2,4(3*H*)-dione (**4f**)


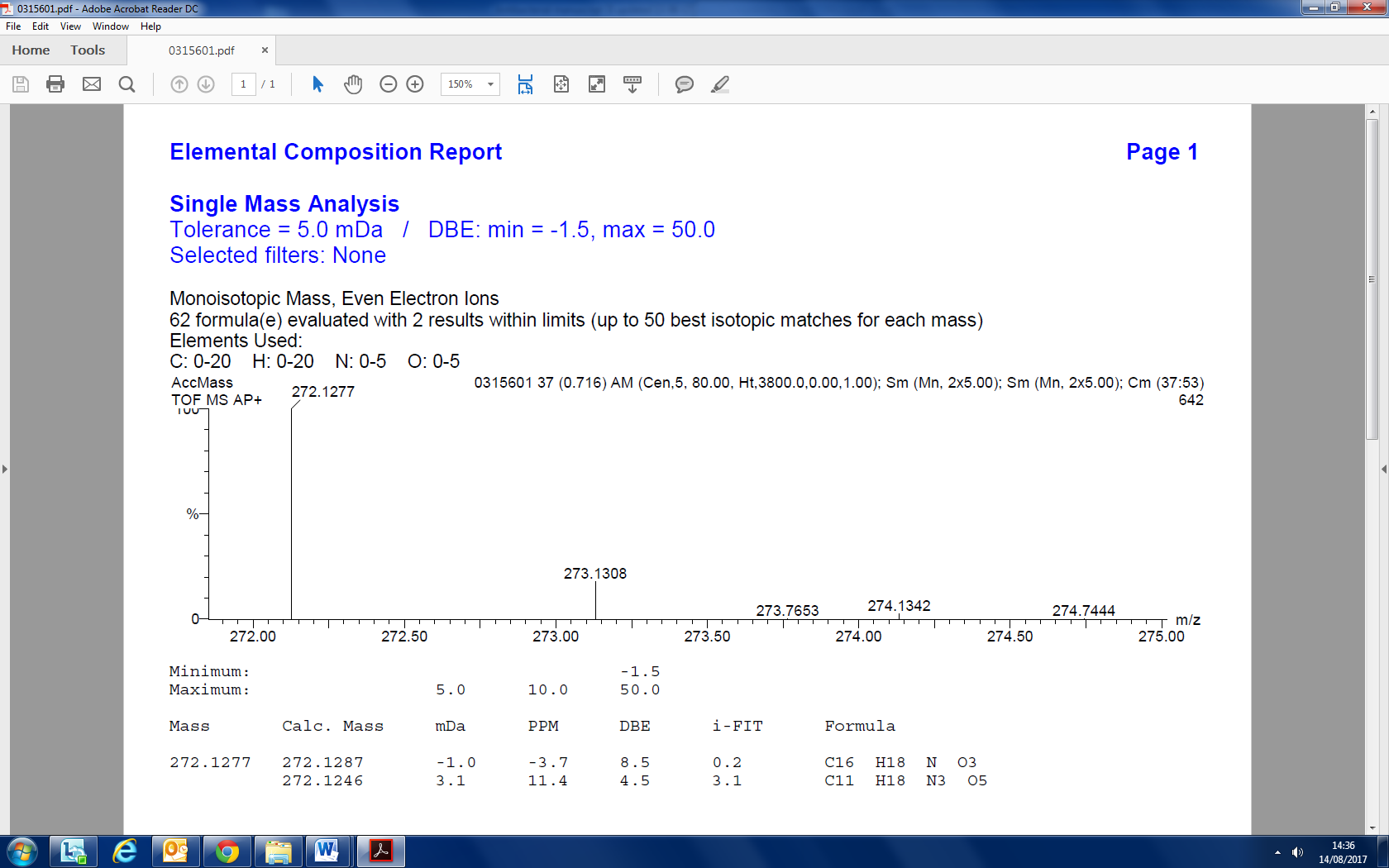


# HRMS(APCI^+^ TOF-MS) of (*E*)-6-methyl-3-(1-(phenethylamino)ethylidene)-2*H*-pyran-2,4(3*H*)-dione (**4f**)


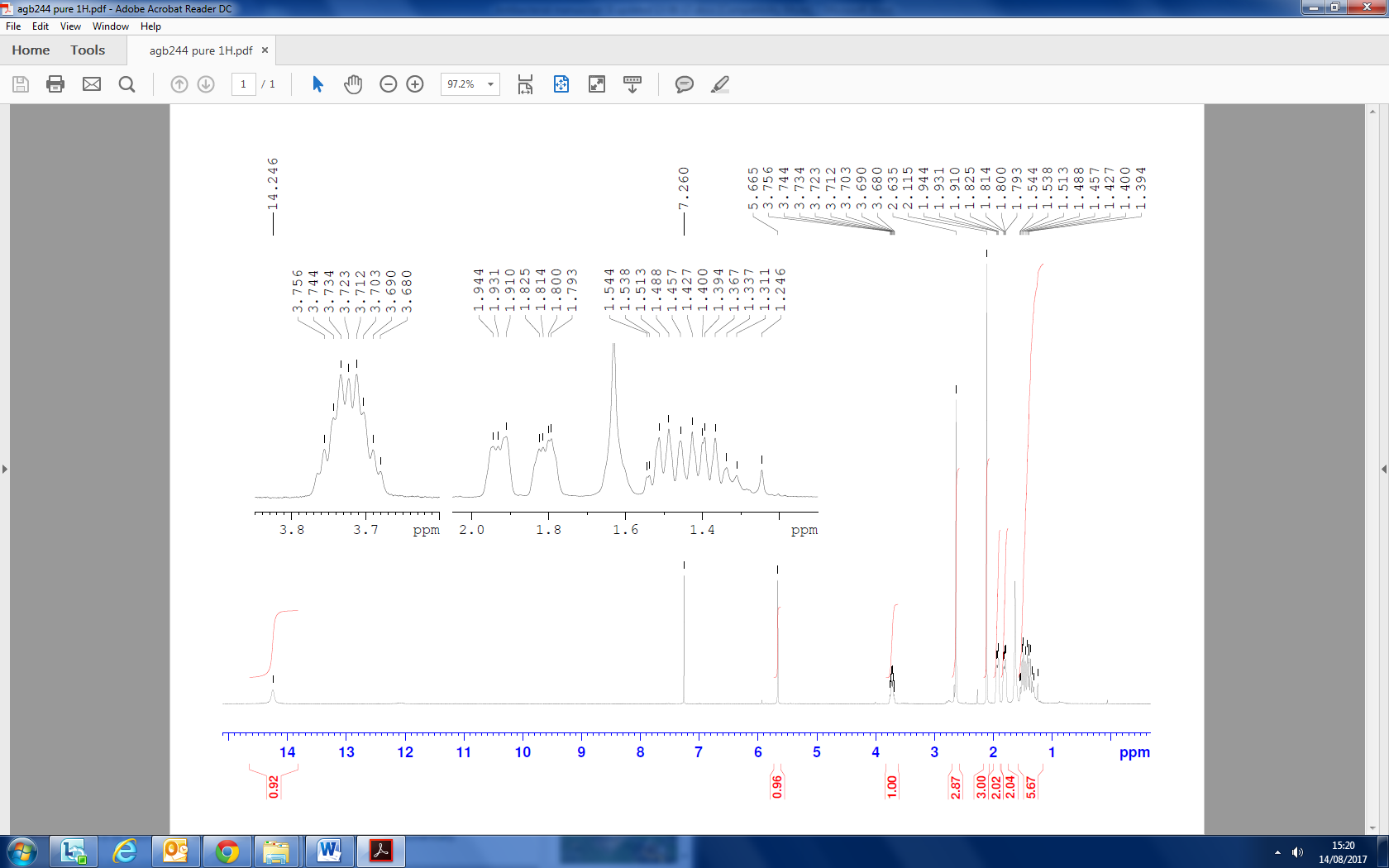


7

H2_ax_/H3_ax_/H4/H5_ax_/H6_ax_

9

H2’_eq_/H6’_eq_

H3’_eq_/H5’_eq_

H_2_O

CHCl_3_

5

1’

NH

# ^1^H NMR spectrum (CDCl_3_) of (*E*)-3-(1-(cyclohexylamino)ethylidene)-6-methyl-2*H*-pyran-2,4(3*H*)-dione (**4g**)


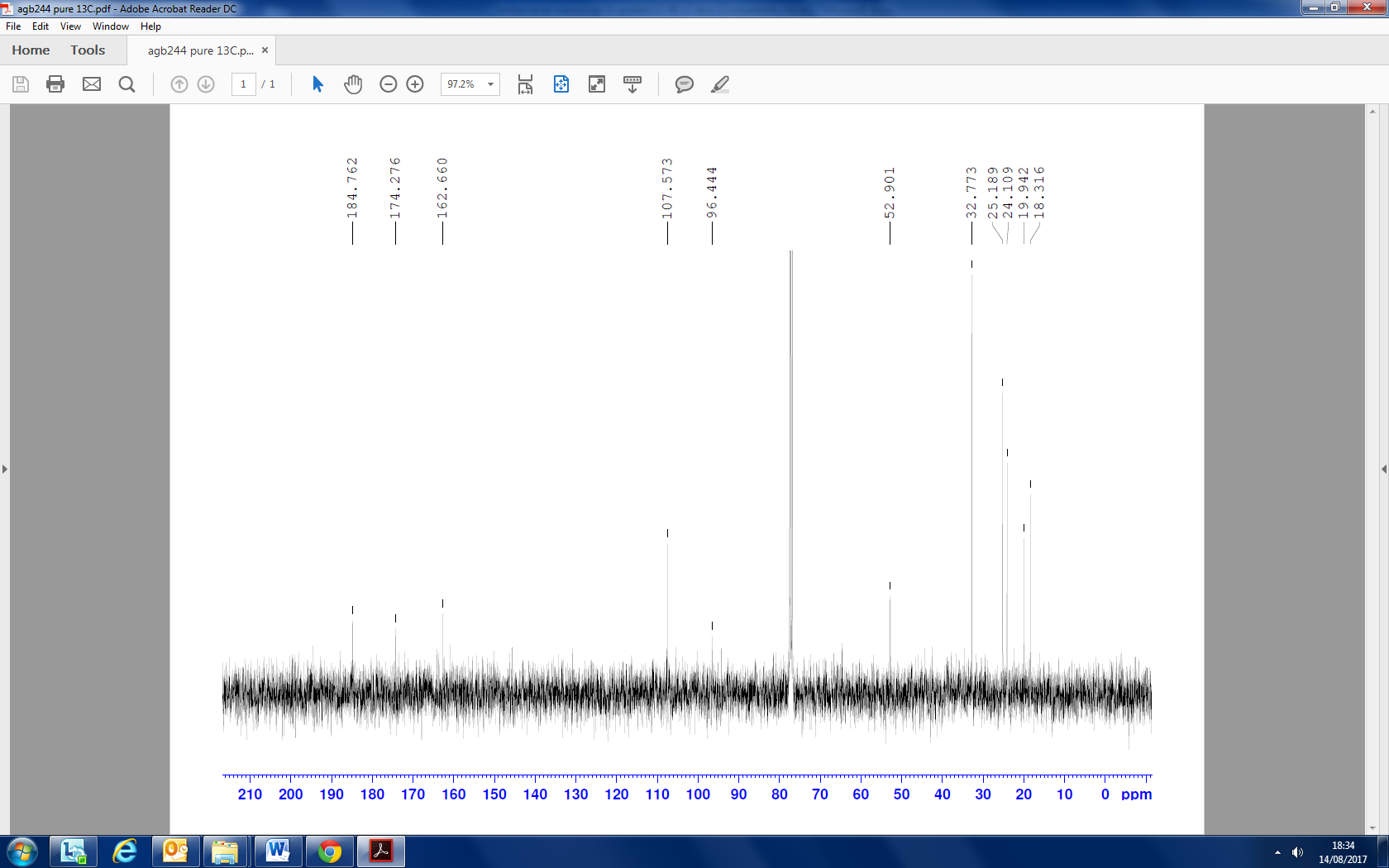


CHCl_3_

2’/6’

3’/5’

4’

7

9

5

1’

6

8

3

4

# ^13^C NMR spectrum (CDCl_3_) of (*E*)-3-(1-(cyclohexylamino)ethylidene)-6-methyl-2*H*-pyran-2,4(3*H*)-dione (**4g**)


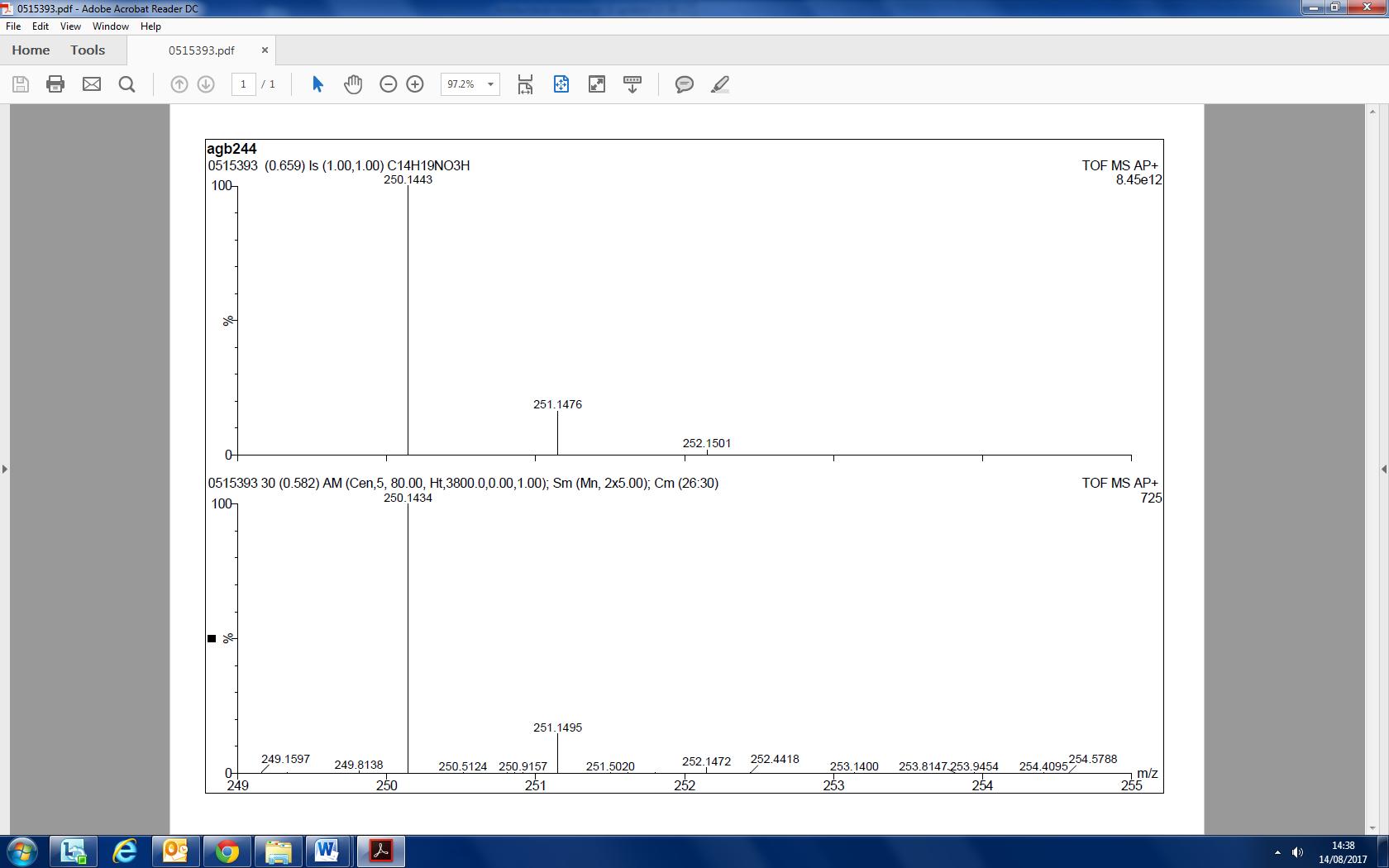


# HRMS(APCI^+^ TOF-MS) of (*E*)-3-(1-(cyclohexylamino)ethylidene)-6-methyl-2*H*-pyran-2,4(3*H*)-dione (**4g**)

^
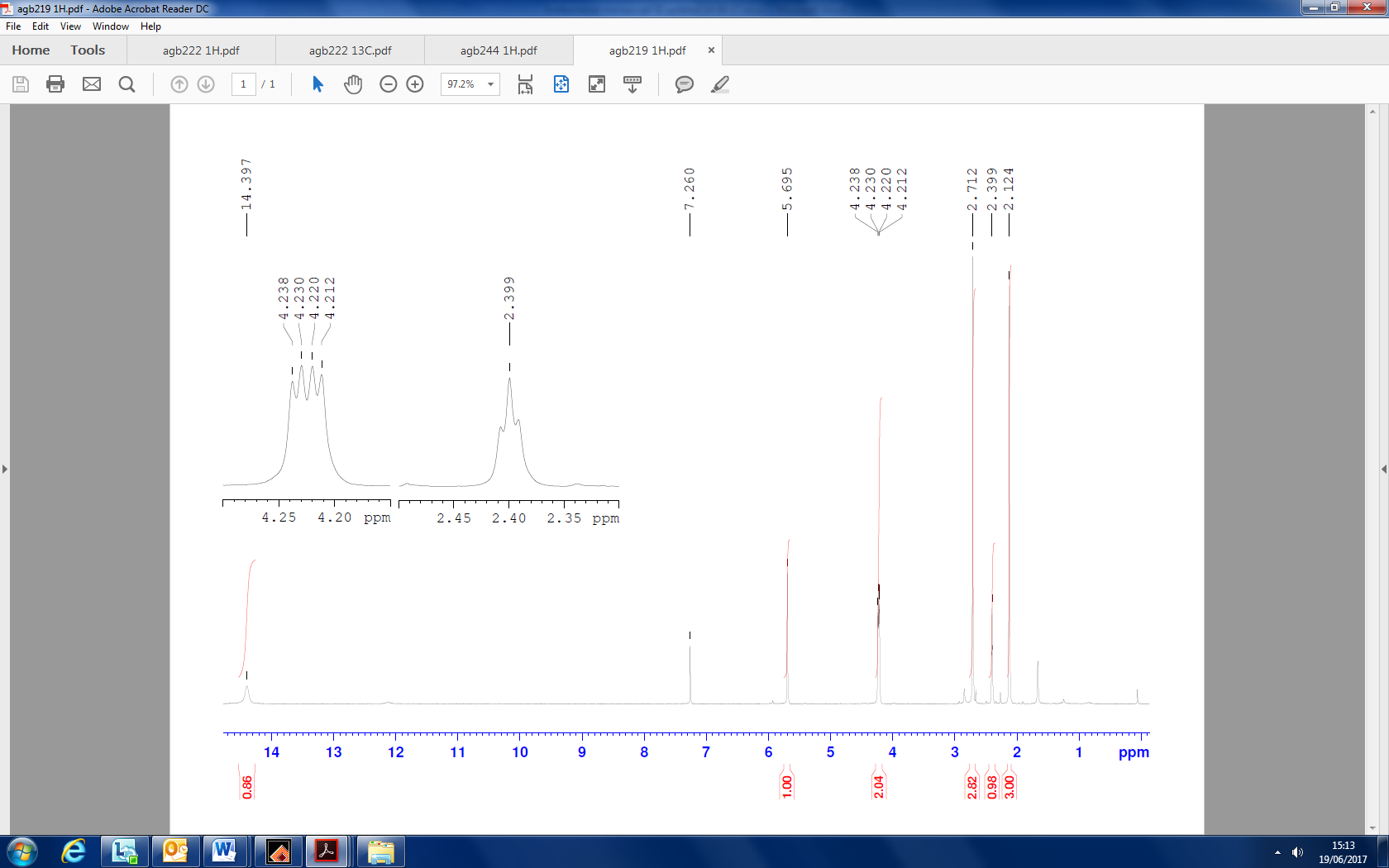
^

9

7

1’

5

3’

NH

CHCl_3_

H_2_O

# ^1^H NMR spectrum (CDCl_3_) of (*E*)-6-methyl-3-(1-(prop-2-yn-1-ylamino)ethylidene)-2*H*-pyran-2,4(3*H*)-dione (**4h**)


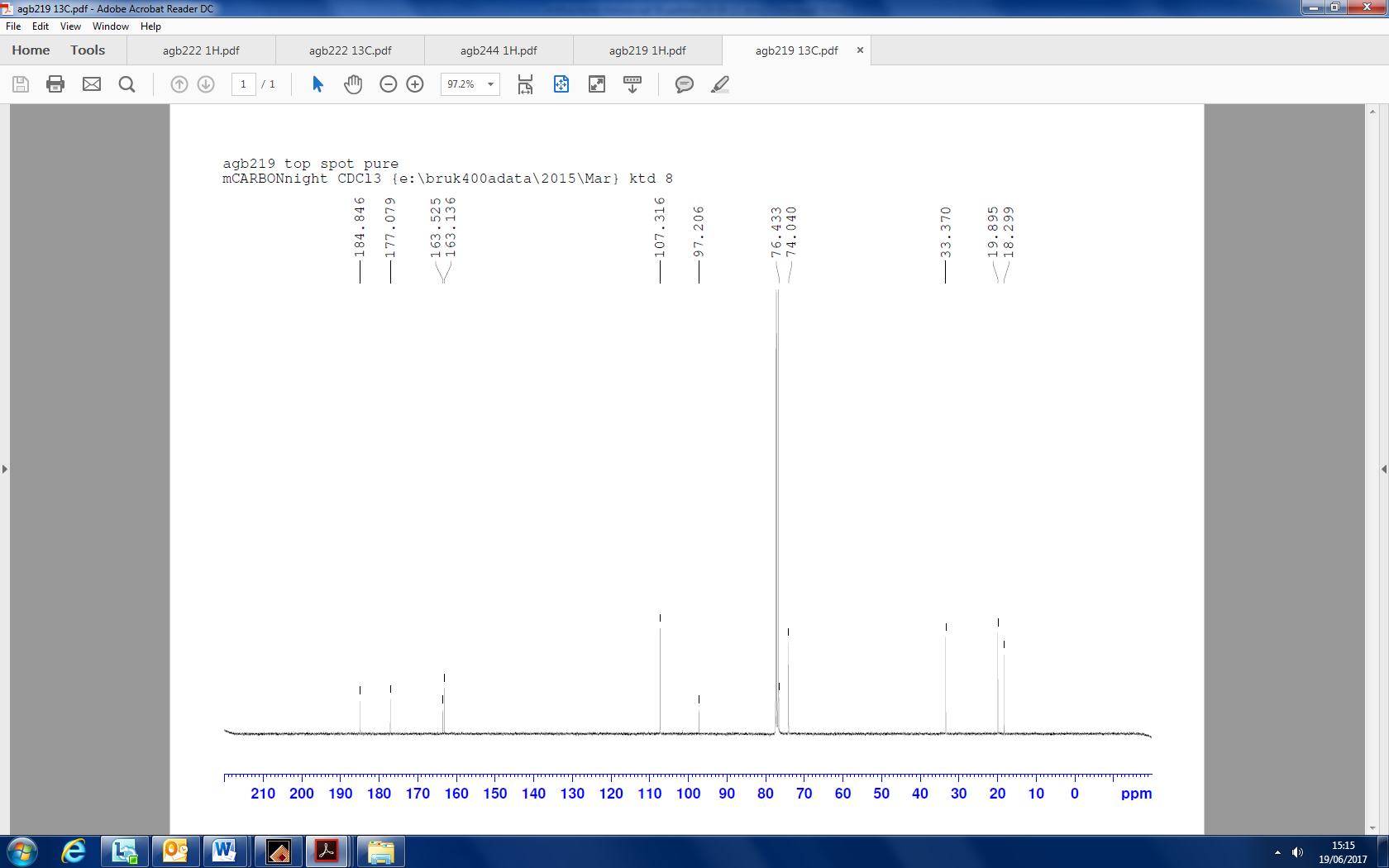

CHCl_3_

5

7

9

1’

3’

6

8

2’

3

2

4

# ^13^C NMR spectrum (CDCl_3_) of (*E*)-6-methyl-3-(1-(prop-2-yn-1-ylamino)ethylidene)-2*H*-pyran-2,4(3*H*)-dione (**4h**)


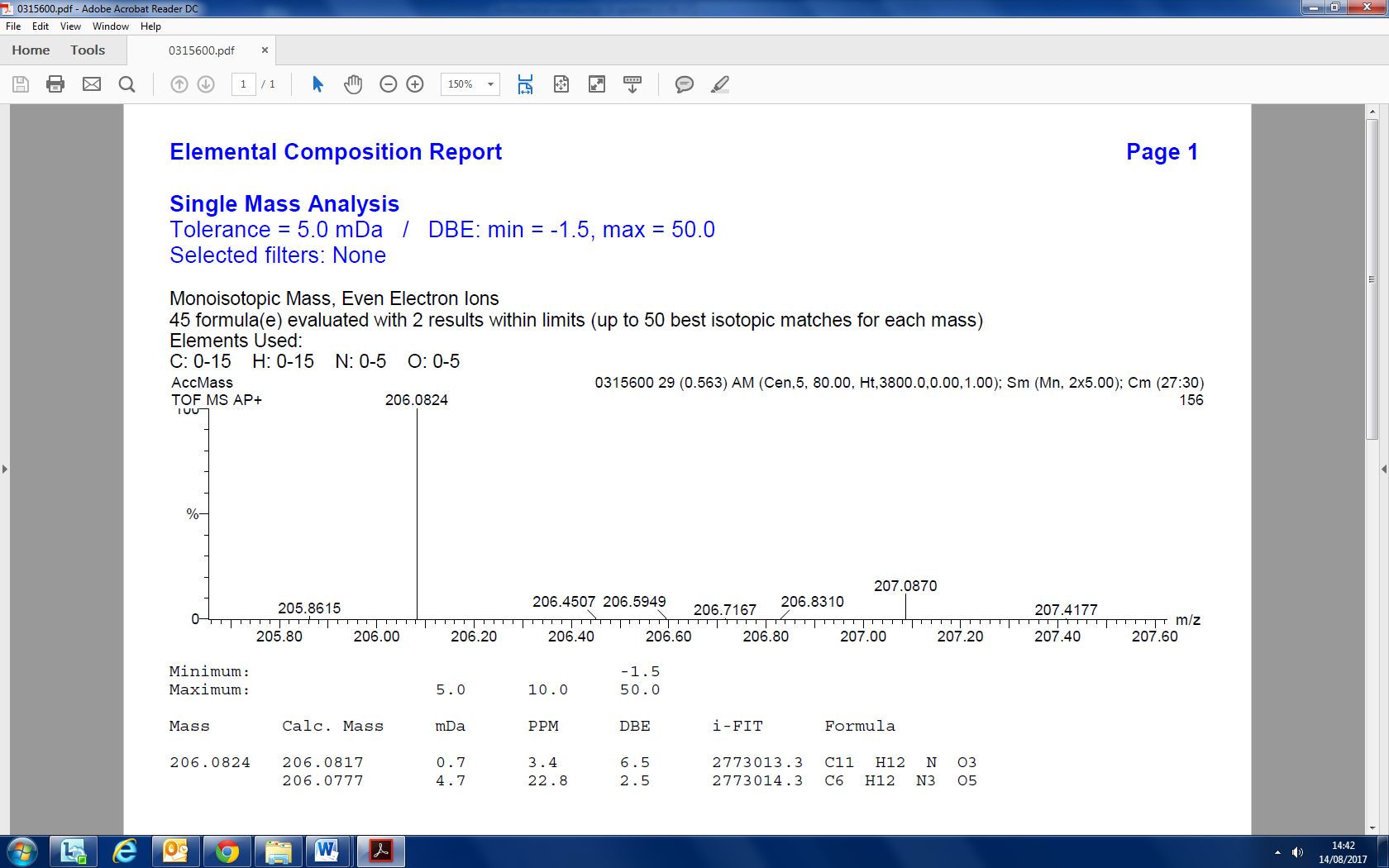


# HRMS(APCI^+^ TOF-MS) of (*E*)-6-methyl-3-(1-(prop-2-yn-1-ylamino)ethylidene)-2*H*-pyran-2,4(3*H*)-dione (**4h**)

^
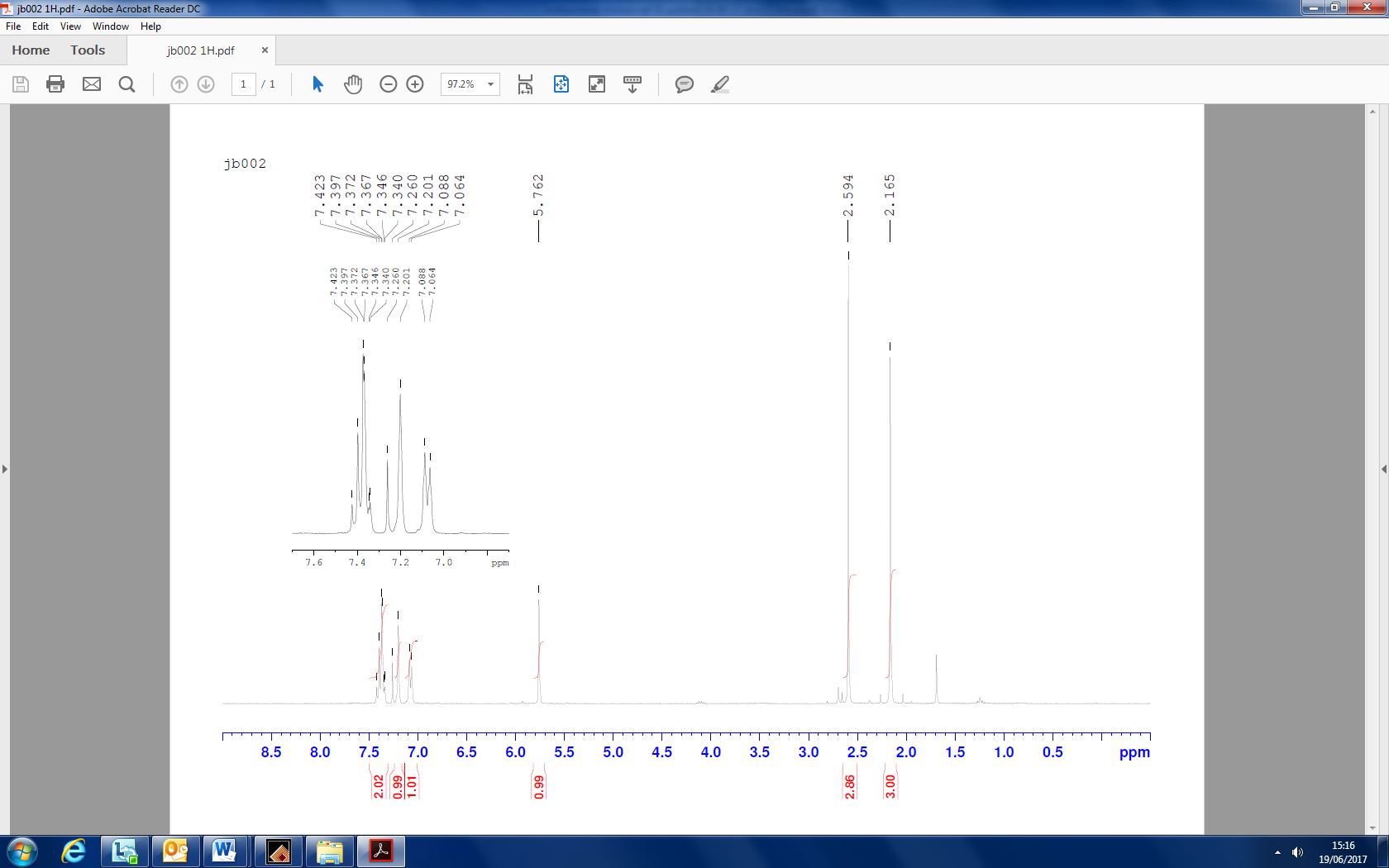
^

9

7

4’/5’

2’

# ^
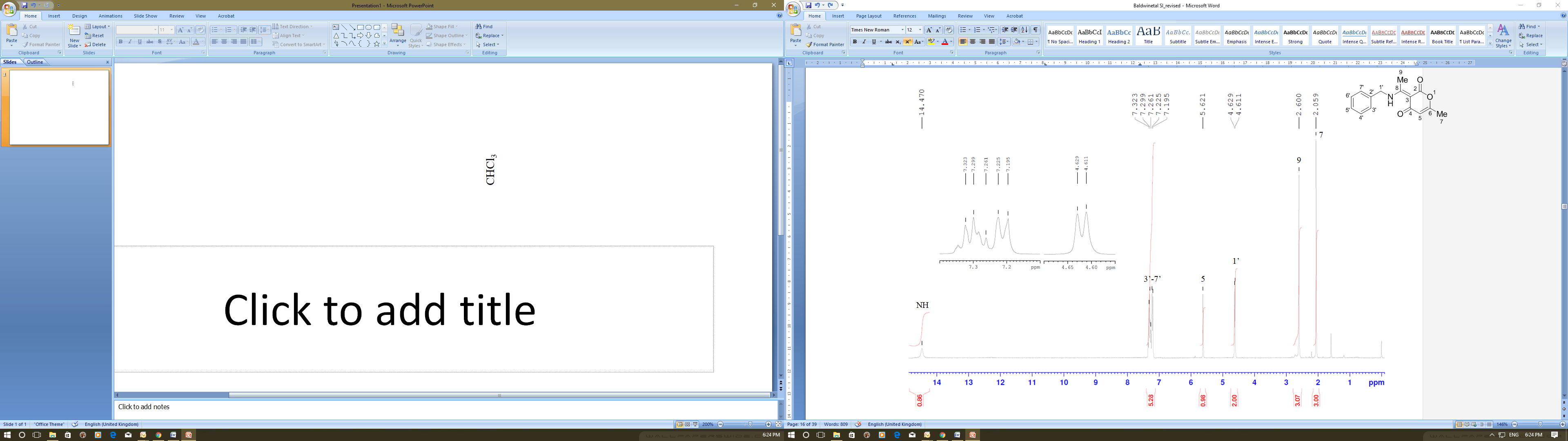
^

6’

5

H_2_O

# ^1^H NMR spectrum (CDCl_3_) of (*E*)-3-(1-(3-chlorophenylamino)ethylidene)-6-methyl-2*H*-pyran-2,4(3*H*)-dione (**4i**)


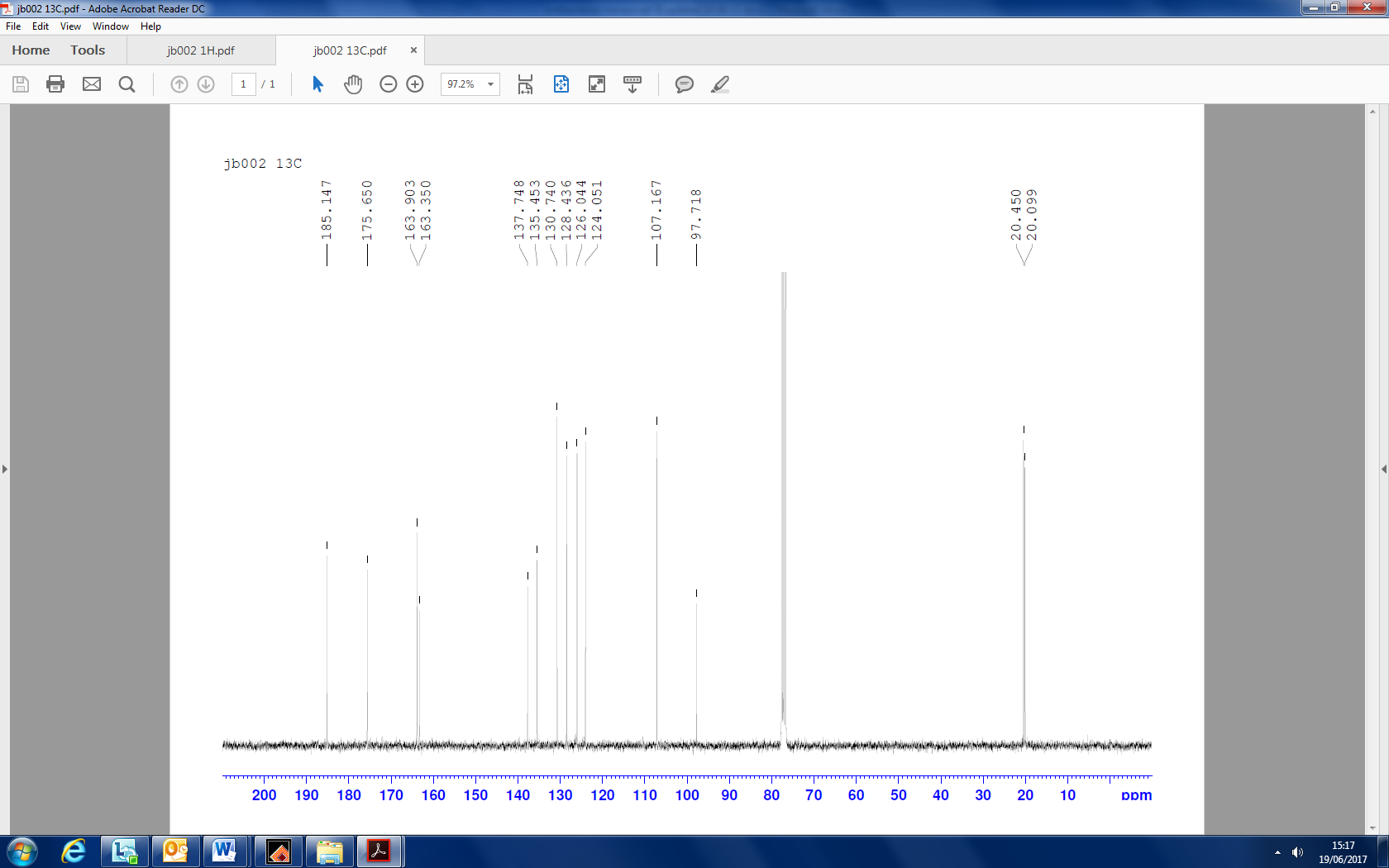


CHCl_3_

5

5’

6’

9

2’

4’

7

2

3’

4

8

3

1’

6

# ^13^C NMR spectrum (CDCl_3_) of (*E*)-3-(1-(3-chlorophenylamino)ethylidene)-6-methyl-2*H*-pyran-2,4(3*H*)-dione (**4i**)

# HRMS(ESI^+^) of (*E*)-3-(1-(3-chlorophenylamino)ethylidene)-6-methyl-2*H*-pyran-2,4(3*H*)-dione (**4i**)


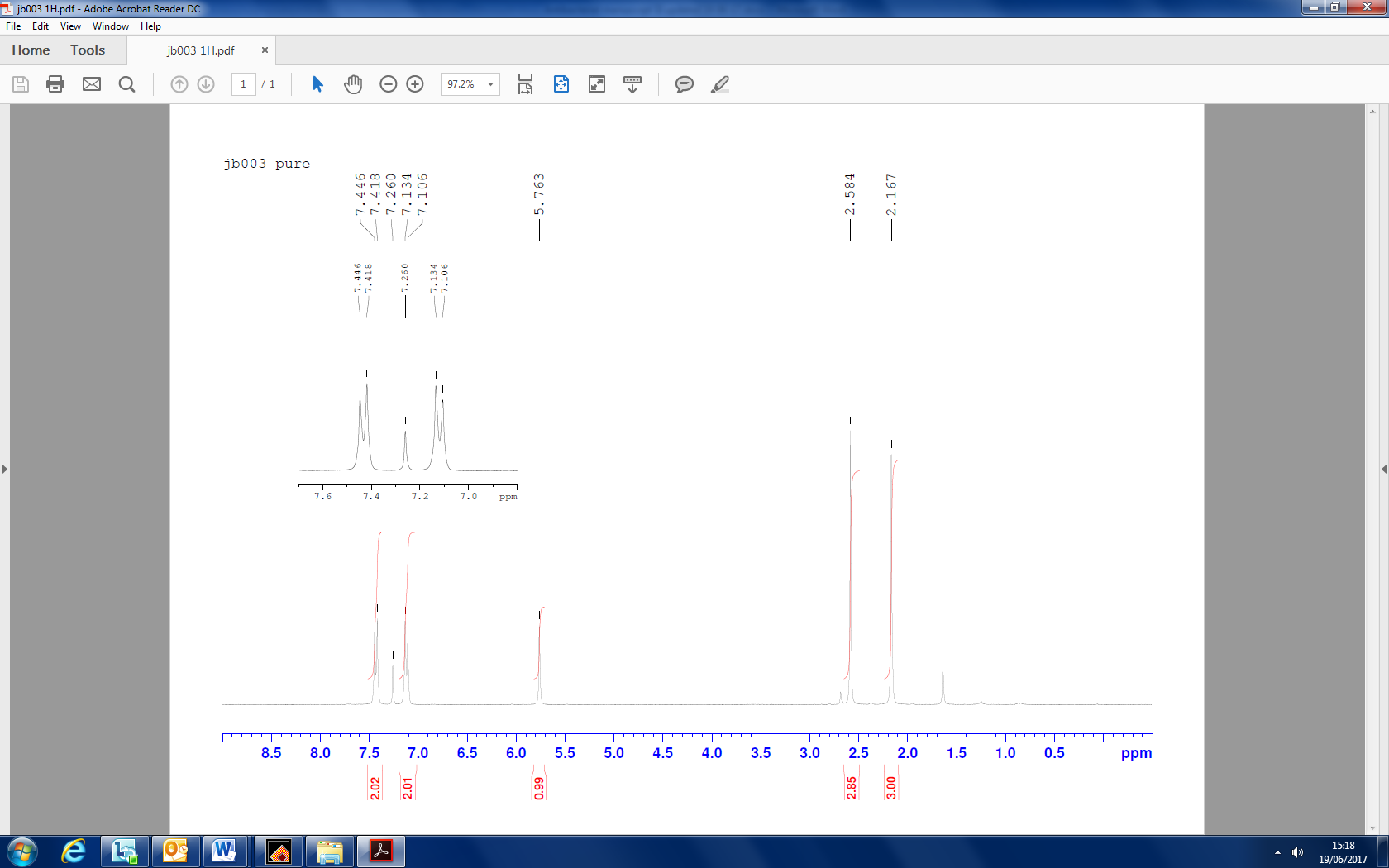

3’/5’

2’/6’

CHCl_3_

9

7

5

H_2_O

# ^1^H NMR spectrum (CDCl_3_) of (*E*)-3-(1-(4-chlorophenylamino)ethylidene)-6-methyl-2*H*-pyran-2,4(3*H*)-dione (**4j**)


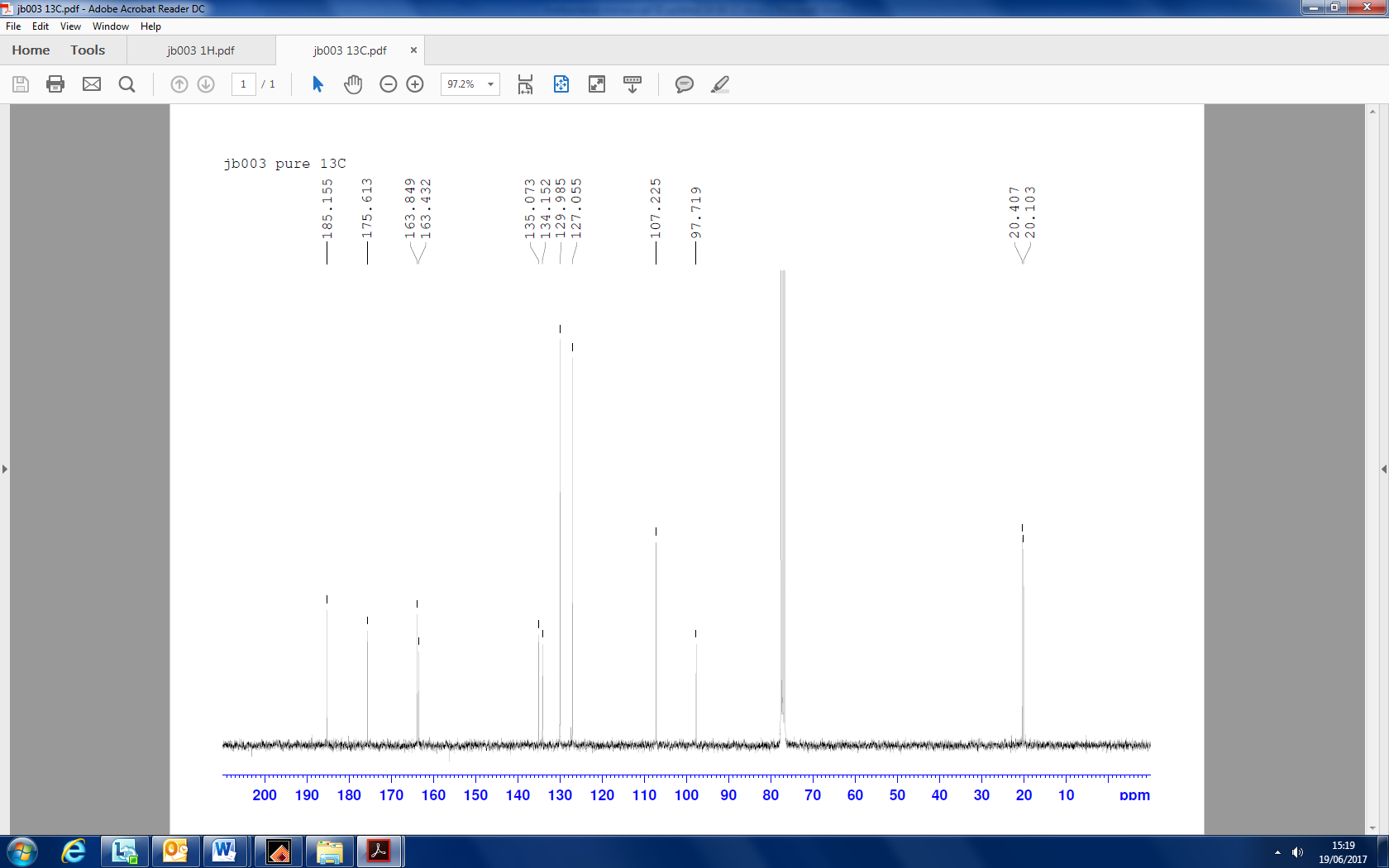


CHCl_3_

2’/6’

3’/5’

5

9

7

4’

3

1’

2

4

8

6

# ^13^C NMR spectrum (CDCl_3_) of (*E*)-3-(1-(4-chlorophenylamino)ethylidene)-6-methyl-2*H*-pyran-2,4(3*H*)-dione (**4j**)

# HRMS(ESI^+^) of (*E*)-3-(1-(4-chlorophenylamino)ethylidene)-6-methyl-2*H*-pyran-2,4(3*H*)-dione (**4j**)

^
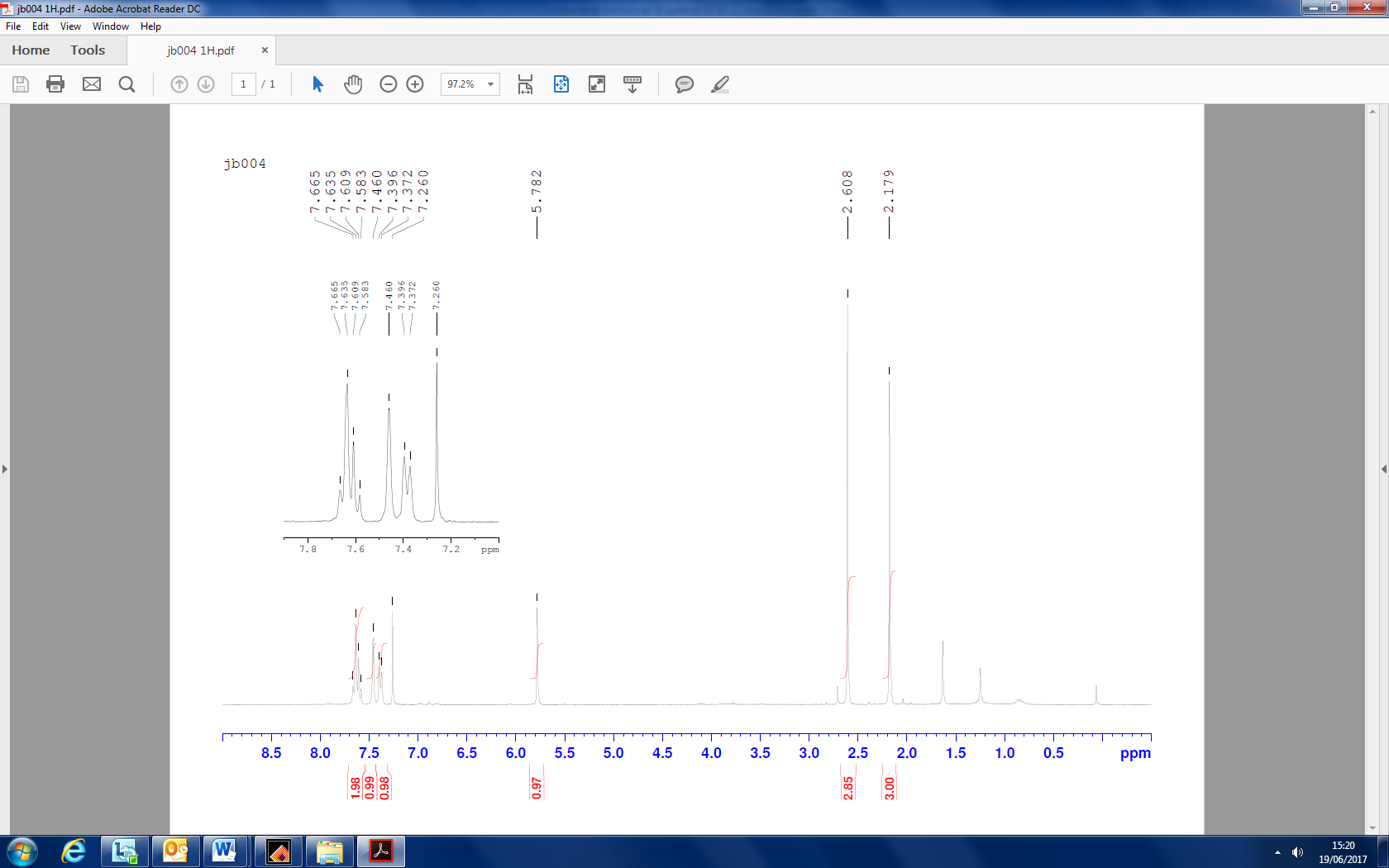
^

9

7

4’/5’

2’

6’

CHCl_3_

5

H_2_O

# ^1^H NMR spectrum (CDCl_3_) of (*E*)-6-methyl-3-(1-((3-(trifluoromethyl)phenyl)amino)ethylidene)-2*H*-pyran-2,4(3*H*)-dione (**4k**)


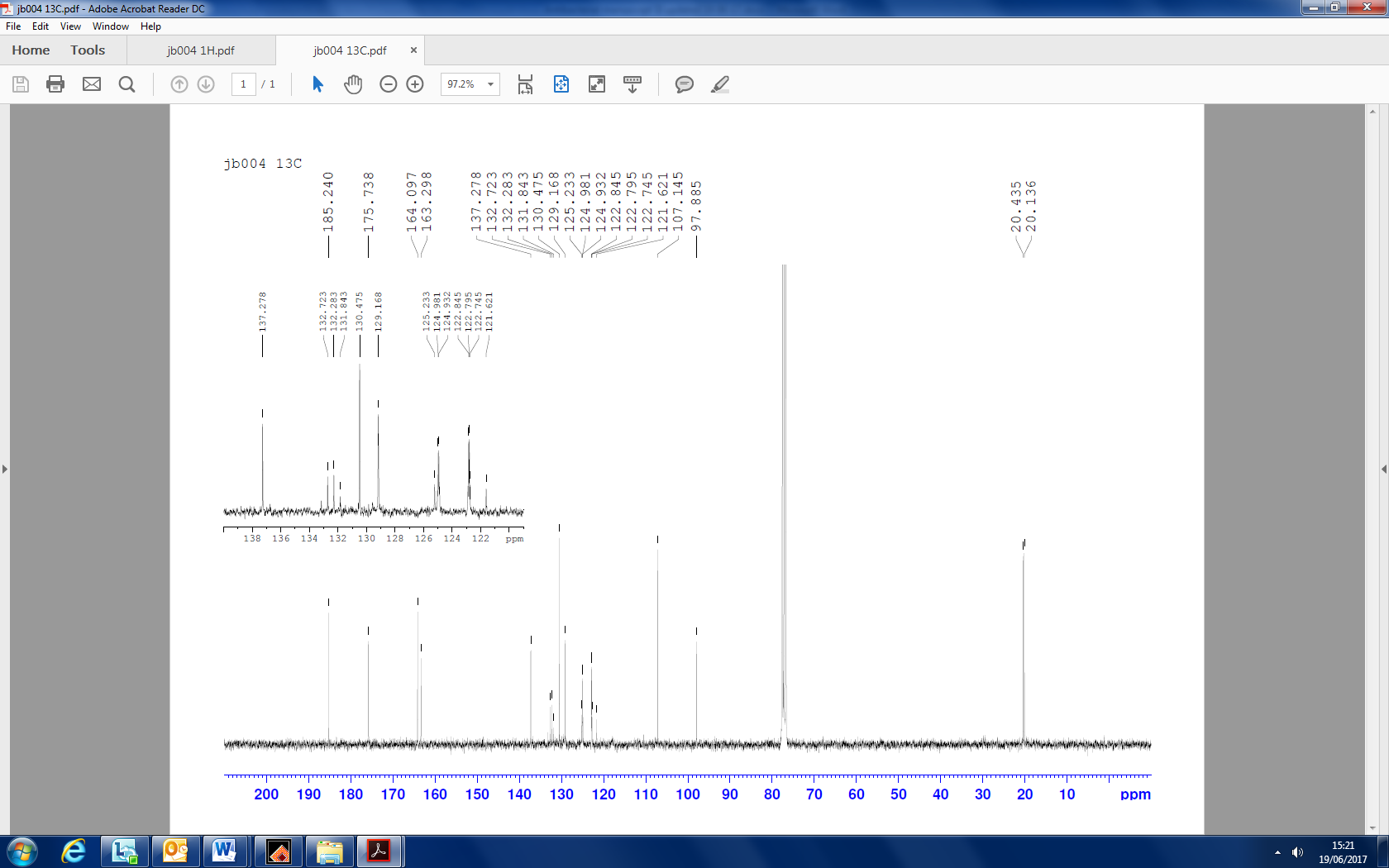


CHCl_3_

7’

5’

4’

2’

6’

1’

3’

7

9

5

3

2

4

8

6

# ^13^C NMR spectrum (CDCl_3_) of (*E*)-6-methyl-3-(1-((3-(trifluoromethyl)phenyl)amino)ethylidene)-2*H*-pyran-2,4(3*H*)-dione (**4k**)

# HRMS(ESI^+^) of (*E*)-6-methyl-3-(1-((3-(trifluoromethyl)phenyl)amino)ethylidene)-2*H*-pyran-2,4(3*H*)-dione (**4k**)


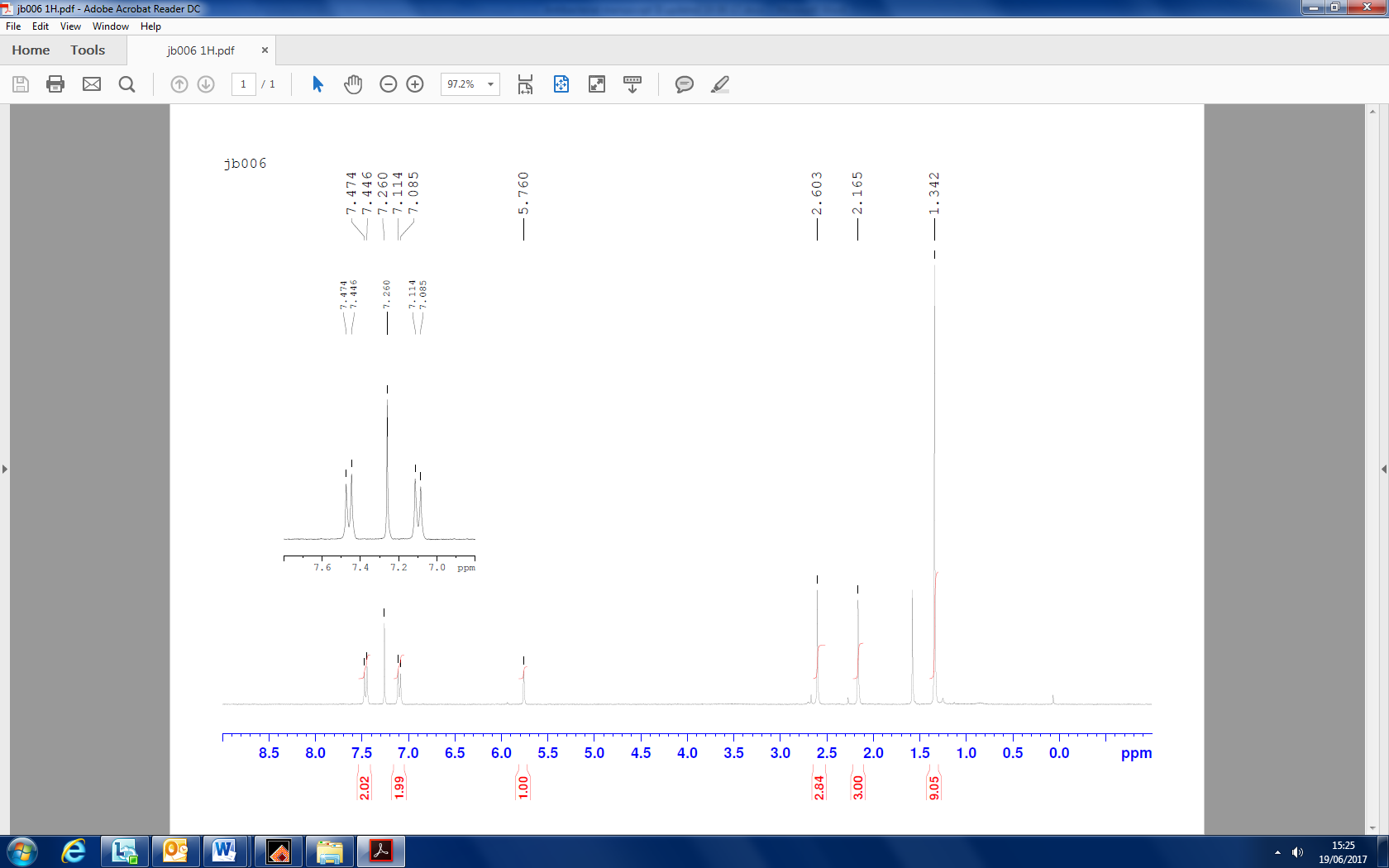


8’

CHCl_3_

2’/6’

3’/5’

H_2_O

7

9

5

# ^1^H NMR spectrum (CDCl_3_) of (*E*)-3-(1-((4-(*tert*-butyl)phenyl)amino)ethylidene)-6-methyl-2*H*-pyran-2,4(3*H*)-dione (**4l**)


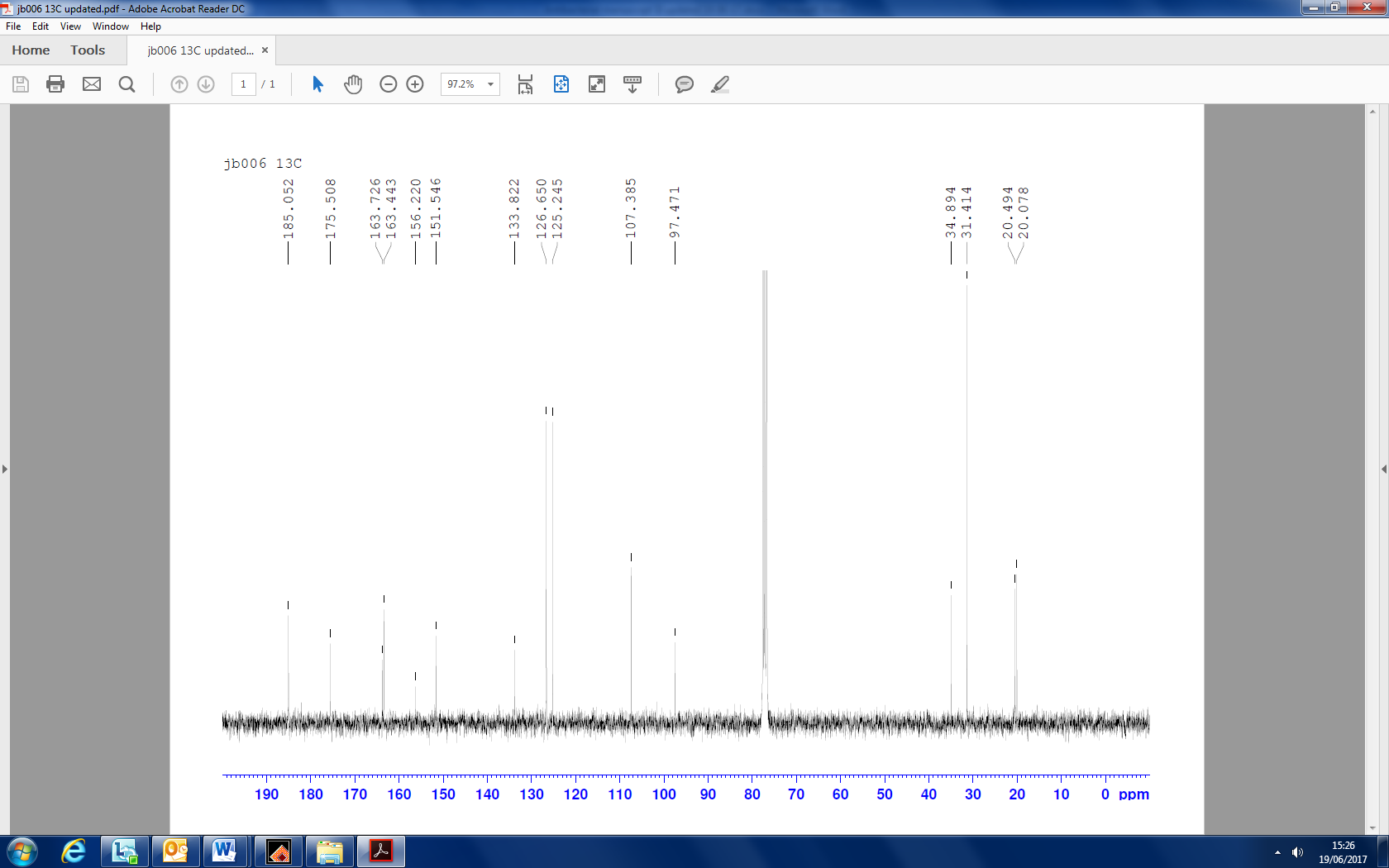


CHCl_3_

8’

2’/6’

3’/5’

5

9

7

7’

3

4’

6

4

8

1’

2

# ^13^C NMR spectrum (CDCl_3_) of (*E*)-3-(1-((4-(*tert*-butyl)phenyl)amino)ethylidene)-6-methyl-2*H*-pyran-2,4(3*H*)-dione (**4l**)

# HRMS(ESI^+^) of (*E*)-3-(1-((4-(*tert*-butyl)phenyl)amino)ethylidene)-6-methyl-2*H*-pyran-2,4(3*H*)-dione (**4l**)
